# Hepatic Transcriptome Responses in Mice (*Mus musculus*) Exposed to the Nafion Membrane and Its Combustion Products

**DOI:** 10.1371/journal.pone.0128591

**Published:** 2015-06-09

**Authors:** Mingbao Feng, Ruijuan Qu, Mussie Habteselassie, Jun Wu, Shaogui Yang, Ping Sun, Qingguo Huang, Zunyao Wang

**Affiliations:** 1 State Key Laboratory of Pollution Control and Resources Reuse, School of the Environment, Nanjing University, Nanjing 210023, P. R. China; 2 College of Agricultural and Environmental Sciences, Department of Crop and Soil Sciences, University of Georgia, Griffin, Georgia 30223, United States of America; 3 College of Resources and Environmental Science, Nanjing Agriculture University, Nanjing 210046, P. R. China; University of Navarra School of Medicine and Center for Applied Medical Research (CIMA), SPAIN

## Abstract

Nafion 117 membrane (N117), an important polymer electrolyte membrane (PEM), has been widely used for numerous chemical technologies. Despite its increasing production and use, the toxicity data for N117 and its combustion products remain lacking. Toxicity studies are necessary to avoid problems related to waste disposal in landfills and incineration that may arise. In this study, we investigated the histopathological alterations, oxidative stress biomarker responses, and transcriptome profiles in the liver of male mice exposed to N117 and its combustion products for 24 days. An ion-chromatography system and liquid chromatography system coupled to a hybrid quadrupole time-of-flight mass spectrometry were used to analyze the chemical compositions of these combustion products. The transcriptomics analysis identified several significantly altered molecular pathways, including the metabolism of xenobiotics, carbohydrates and lipids; signal transduction; cellular processes; immune system; and signaling molecules and interaction. These studies provide preliminary data for the potential toxicity of N117 and its combustion products on living organisms and may fill the information gaps in the toxicity databases for the currently used PEMs.

## Introduction

During the last few decades, the polymer electrolyte membrane (PEM) has gained worldwide attention because of its wide use in a variety of chemical technologies, including for fuel cells, electrodialyzers, and sensors [1,2]. In particular, its application in fuel cells has attracted considerable interest because this technology has recently been used to supply energy to automobiles, for stationary power generation and for portable electronic devices [3,4]. Nafion, a polyperfluorosulfonic acid (PFSA) membrane developed by DuPont, is an important PEM because of its high proton conductivity, good chemical stability and high mechanical strength [5–7]. It consists of a carbon-fluorine backbone with perfluoro side chains containing sulfonic acid groups. In light of the rapid development of the related technologies, the production and use of Nafion is expected to increase rapidly in the future. The disposal of Nafion wastes will therefore become more of a challenge, particularly because landfills and incineration are two common methods used for Nafion waste disposal [8].

Despite the widespread applications of Nafion in various technologies, recent studies focused primarily on its chemical degradation in fuel cells [9,10] and utilization in modified electrodes [11,12] as well as the removal of some pollutants [13,14], whereas the related toxicological data are lacking. The toxicity data provided by DuPont indicated an LD_50_ value greater than 20,000 mg/kg body weight in rats and no skin irritation to rabbits and humans [8]. It can be concluded that the potential toxic effects of Nafion are low. However, when Nafion is disposed via incineration, the toxicity of its combustion products may be increased because of the generation of fluorine ions (F^-^) and/or low-molecular-weight organofluorine compounds (S1 Table) [8]. To date, there are no toxicity data on these combustion products; therefore, additional studies are necessary to determine the better utilization and management of Nafion.

Recently, microarray-based toxicogenomics analysis has been used to evaluate the impacts of environmental toxicants on multiple cellular pathways [15–17]. This approach allows the identification of broad-scale gene expression patterns, which allows for the understanding of specific molecular responses and provides useful information on the mechanism of action of the toxicants [15]. However, this approach has not been used to assess the toxic effects of Nafion and its combustion products.

The objective of this study was to evaluate the potential toxicity of the Nafion 117 membrane (N117), a widely used Nafion membrane, and its combustion products on the mouse liver. Mice were exposed to three differentially treated N117 membranes for 24 days, and the histopathological alterations, oxidative stress responses, and transcriptome profiles were determined. Systematic analyses were conducted to identify the molecular pathways related to their potential toxicity. To the best of our knowledge, this is the first detailed assessment of the biotoxicity of N117 and its combustion products.

## Materials and Methods

### Ethics statement

All experiments involving animals were conducted in accordance with the National Institutes of Health (NIH) Guide for the Care and Use of Laboratory Animals (NIH publication number 85–23, revised 1996). All procedures were approved by the Institutional Animal Care and Use Committee of Model Animal Research Center of Nanjing University. Remarkable efforts were made to minimize the number of animals used in the study and their sufferings. All animals were housed in stainless-steel cages and acclimated for one week with regulated temperature (22 ± 1°C) and humidity (55 ± 5%), a 12/12 h light/dark cycle, and free access to food and water. At the end of the treatments, mice were fasted over night, anaesthetized with diethyl ether, and killed by cervical dislocation.

### N117 treatment

To better mimic the actual exposure conditions of landfills or incineration sites, N117 (Shanghai Hesen Electric Co., Ltd, Shanghai, China) was cut into small pieces (1.0 mm × 1.0 mm) and then subjected to the following three different treatments: 1) directly mixed with mouse food; 2) combusted in the absence of oxygen with the products absorbed into the water to feed the mice; and 3) combusted in the presence of oxygen with the products absorbed into water to feed the mice. The details of the experimental treatments are described below.

1. Mixing of N117 with mouse food (1/100 wt% for N117/food): Mouse food, purchased from Qinglongshan Animal Breeding Center (Nanjing, China), was ground into a powder with mortar and pestle and then mixed with N117 pieces. After adequate mixing, a small amount of drinking water (30% v/w) was added to shape the mixture, which was then dried in an oven overnight at 103°C for the subsequent feeding test.
2. Combustion lacking oxygen supplementation (CLOS; 100 mg N117/L): Small pieces of the N117 (1 g) were heated in specialized pyrolysis equipment (AZ-HC-06a, Tianjin Aozhan Technology Co., Ltd, Tianjin, China). The operating parameters, including the atmosphere, heating temperature and time, were set according to the literature [8]. Specifically, the atmosphere was air, and the flow rate was 13 mL/min. The N117 sample was heated in a stainless steel tube at 10°C/min to 200°C and then for 5°C/min to 400°C; the temperature was then held for an additional 20 minutes for a total run time of approximately 75 minutes. The combustion products were absorbed with a 0.05 M sodium hydroxide (NaOH, 1 L) solution. The solution was then adjusted to pH 7.0 using 0.1 M hydrochloric acid, diluted 10-fold with drinking water and stored for the subsequent drinking test.
3. Oxygen-enriched combustion (OEC; 100 mg N117/L): Small pieces of the N117 (0.05 g) were ignited in a conical flask using the oxygen flask combustion (OFC) method (S1 File) [18,19], and the combustion products were absorbed with a 0.05 M NaOH solution (0.05 L). The solution was then adjusted to pH 7.0 using 0.1 M hydrochloric acid, diluted 10-fold with drinking water and stored for the subsequent drinking test.

### Animal exposure

Twenty-four male mice (*Mus musculus*) (18–20 g), five-weeks of age, were purchased from Qinglongshan Animal Breeding Center (Nanjing, China). The mice were housed in stainless-steel cages and acclimated for one week with regulated temperature (22 ± 1°C) and humidity (55 ± 5%), a 12/12 h light/dark cycle, and free access to food and water. The mice were randomly divided into one control and three N117-treated groups, with six mice in each group. For the control group, a normal diet was provided for the mice daily. For the three experimental groups, one group received the N117-treated food and normal drinking water, and the other two groups received the normal food and treated water. They were exposed to these treatments for 24 days and named the Food group, CLOS group, and OEC group, respectively. After the exposure, all mice after diethyl ether anaesthesia were sacrificed by cervical dislocation for tissue collection.

### F^-^ determination and LC/MS analysis

The tissue samples (liver, kidney, and muscle) were dissected from the mice, freeze-dried for 48 h with a Labconco Freeze Dry System (Labconco, Kansas City, MO, USA) and manually ground into powders, which were further passed through 0.4 × 0.4 mm screen. To quantify the fluorine concentration in the tissue samples, 0.05 g of each sample was first combusted using the OFC method with the gases absorbed in 50 mL 0.05 M NaOH solution. The solution was then adjusted to pH 7.0 using hydrochloric acid, followed by F^-^ analysis using a fluoride ion selective electrode (Shanghai Precision Scientific Instrument Co. Ltd., Shanghai, China) [18]. The F^-^ concentrations in the mouse urine were also measured with this electrode. For the drinking water samples, the F^-^ concentrations were determined using an ion-chromatograph system (ICS-1000, Dionex, USA) (S2 File).

The product composition of the initial absorption solutions of the different N117 combustion treatments was analyzed using liquid chromatography coupled with a high-resolution hybrid quadrupole time-of-flight mass spectrometry LCMS-Q-TOF (LCMS-Triple TOF 5600, AB SCIEX, Foster City, CA) (S3 File).

### Histopathological analysis

The liver tissue was harvested and fixed in 10% formalin, washed and dehydrated with a graded series of ethanol, and embedded in paraffin blocks. Sections of 5 *μ*m thickness were stained with hematoxylin-eosin (H&E) and observed under a light microscope.

### Oxidative stress analysis and integrated biomarker response

The liver samples were homogenized and centrifuged, and the supernatants were collected for biomarker determination. The biochemical parameters for oxidative stress, including the activities of superoxide dismutase (SOD) [20] and catalase (CAT) [21], reduced glutathione (GSH) content [22], and level of the lipid peroxidation product malondialdehyde (MDA) [23], were measured using commercial kits (Nanjing Jiancheng Bioengineering Institute, Nanjing, China) and normalized by protein content, which was determined by Bradford method [24]. Each experiment was performed in triplicate.

A method for combining all of the measured biomarker signals into one integral “stress index”, termed the “Integrated Biomarker Response” (IBR) (S4 File) [25,26] was used to evaluate and compare the integrated impacts of N117 with different treatments on the antioxidant status of the mouse liver.

### RNA extraction and microarray analysis

Total RNA from each mouse liver was extracted using TRIzol reagent (Invitrogen, Carlsbad, CA, USA) and purified with a RNeasy mini kit (QIAGEN GmBH, Germany). The RNA concentrations were quantified using a NanoDrop ND-1000 spectrophotometer (Thermo Scientific, Wilmington, DE, USA), with the RNA integrity determined as the RNA Integrity Number (RIN) using an Agilent Bioanalyzer 2100 (Agilent Technologies, Santa Clara, CA, USA). RNA samples with a 2100 RIN value ≥ 7 and 28S/18S ≥ 0.7 were used for the gene expression analysis.

Microarray analysis was conducted by the Shanghai Biotech Corp (SBC, Shanghai, China). RNA extracted from one mouse liver, which was randomly selected in each group, was one sample. Four biological samples for all of the treatments were individually applied to the Agilent Whole Mouse Genome Oligo Microarray (4 × 44 K) platform (Agilent Technologies, Santa Clara, CA, USA) containing 41,174 unique probes. The subsequent RNA linear amplification and microarray hybridization were performed following the manufacturer’s instructions. After the hybridization, the microarray slides were scanned with an Agilent Microarray Scanner (Agilent Technologies, Santa Clara, CA, USA) at a 5 *μ*m resolution for each slide with a photomultiplier tube setting of 100% and 10%. The data were extracted with the Feature Extraction software 10.7 (Agilent Technologies, Santa Clara, CA, USA). The raw data were normalized with the Quantile algorithm, Gene Spring Software 11.0 (Agilent Technologies, Santa Clara, CA, USA). Differentially expressed genes (DEGs) between the treated groups and control were identified as the genes with a greater than ± 2.0-fold-change and *p*-value < 0.05 (*t*-test). Gene ontology (GO) analysis (http://www.geneontology.org/) and Kyoto encyclopedia of genes and genomes (KEGG) pathway analysis (http://www.genome.ad.jp/kegg/pathway.html) were performed using the SBC Analysis System of the Shanghai Biotech Corp (http://www.ebioservice.com/).

### Quantitative real-time polymerase chain reaction (QRT-PCR) analysis

Validation of the transcriptomic profiles was performed using QRT-PCR analyses with four target genes (*Ugt1a2*, *Map3k6*, *Ccnb1* and *Ccl5*), which were differentially expressed in liver of mice exposed to N117 and its combustion products for 24 days. The primer sequences and PCR product sizes were shown in S2 Table. QRT-PCR analyses were conducted on all liver samples in each group (S5 File).

### Statistical analysis

The data are expressed as the mean ± standard deviation (SD) and analyzed with the SPSS 16.0 software (SPSS Inc., Chicago, IL, USA). Tests for normal distribution (Kolmogorov-Smirnov) and homogeneity of variances (Levene) were applied. One-way ANOVA followed by Dunnett’s *t* test was performed to determine the significant differences of the biological parameters between the N117-treated groups and control. The results were considered significant at *p* < 0.05 and *p* < 0.01.

## Results

### Body and relative organ weight

Compared to the control, no significant differences (*p* > 0.05) were observed in the body and relative organ weights (liver and kidney) of the mice after a 24-day exposure to three different N117 treatments (S3 Table).

### F^-^ analysis

The F^-^ concentrations in the mouse tissues, urine and drinking water samples in the control and N117-treated groups are listed in Table 1. Significant increases (*p* < 0.01) in the F^-^ concentrations were observed in the CLOS and OEC groups compared to the control. The highest value was found in the liver of the OEC group (1946.24 mg/kg dry weight), which was approximately 5.76 times that of the control. The F^-^ concentrations in the kidney, muscle and urine were approximately 2.45, 3.56, and 6.38 times greater in the OEC group than the control group, whereas a much higher F^-^ concentration was found in the drinking water for the OEC group (41.6 mg/L). The increases in the F^-^ concentrations in the CLOS group were less significant than the OEC group, and the F^-^ concentration in the drinking water for the CLOS group was also lower than the OEC group was more elevated than the control (Table 1). In the Food group, only slight increases (*p* > 0.05) were found in the mouse tissues and urine compared to the control.

**Table 1:** F^-^ concentrations in drinking water samples, mice tissues and urine in the control and N117-treated groups after 24-day exposure.

	Control	Food	CLOS	OEC
Drinking water (mg/L)	0.68±0.05	0.65±0.08	8.45±0.59	41.6±1.51
Liver (mg/kg dry weight)	337.69±27.42	363.79±54.34	643.21±51.77**	1946.24±55.60**
Kidney (mg/kg dry weight)	283.23±49.69	332.88±62.06	424.59±67.68	693.23±65.45**
Muscle (mg/kg dry weight)	282.97±23.61	378.48±61.52	771.19±39.52**	1008.80±38.66**
Urine (ng/L)	34.97±5.95	56.67±6.03	100.67±14.30**	223.00±15.62**

Values were obtained using an ion-chromatograph system (for drinking water) or a fluoride ion selective electrode (for mice tissues and urine). Twenty-four male mice (five-weeks of age) were individually exposed to normal diet (Control), 1/100 wt% N117-treated food (Food), 100 mg N117/L treated by combustion lacking oxygen supplementation (CLOS), and 100 mg N117/L treated by oxygen-enriched combustion (OEC) for 24 days, with six mice in each group. After the exposure, the drinking water samples, mice tissues (liver, kidney and muscle) and urine were assayed for F^-^ concentrations. Data are the mean ± standard deviation (SD), *n* = 6 for each data point.

** *p* < 0.01 indicates a significant difference compared with control using one-way ANOVA with Dunnett’s *t* test.

### LC/MS analysis

As shown in S1 Fig, many significant *m/z* peaks were observed in the absorption solution of the CLOS compared to the pure water, whereas only a few *m/z* peaks were observed in the absorption solution of the OEC compared to the filter paper sample. Notably, many more *m/z* peaks were recorded in the absorption solution of the CLOS than OEC. Furthermore, a more complete combustion of N117 from the OEC treatment led to less products but more F^-^ than that from the CLOS treatment.

### Hepatic histopathology

**Fig 1:**
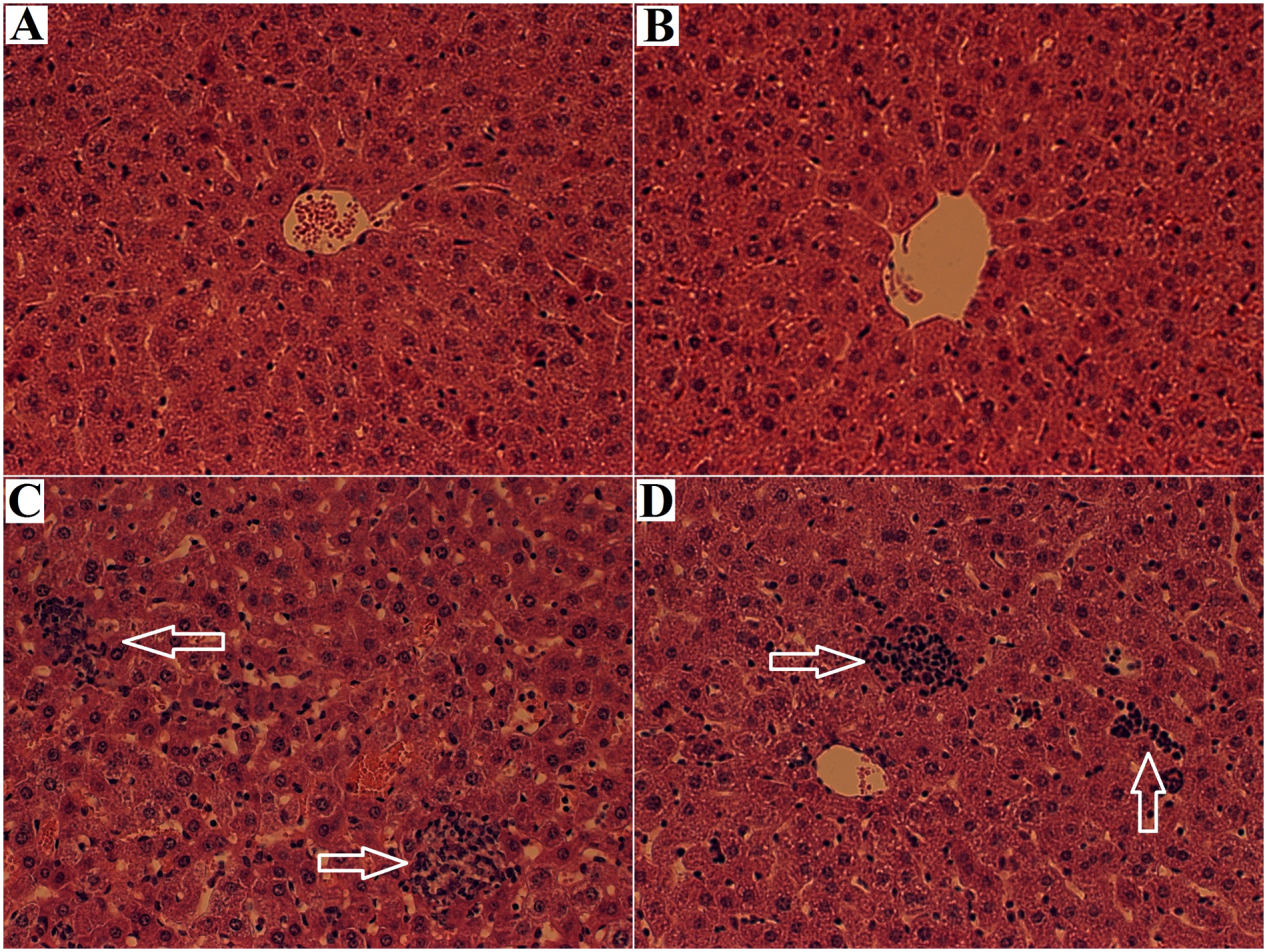
Histopathological alterations in the mouse liver after a 24-day exposure to N117 and its combustion products. Hematoxylin-eosin (H&E) staining performed on 5-*μ*m-thick paraffin-embedded liver sections of male mice (five-weeks of age) individually exposed to normal diet (Control; A), 1/100 wt% N117-treated food (Food; B), 100 mg N117/L treated by combustion lacking oxygen supplementation (CLOS; C), and 100 mg N117/L treated by oxygen-enriched combustion (OEC; D) for 24 days. White arrows indicate the hepatocellular necrosis and inflammation infiltration observed in the CLOS- and OEC-treated mouse liver.

Histopathological alterations in the mouse liver after a 24-day exposure to the three N117 treatments are shown in Fig 1. No obvious effects were observed in the Food group, whereas hepatocellular necrosis and inflammatory infiltration were obvious in the mouse livers of the CLOS and OEC groups.

### Oxidative stress induced by N117 and its combustion products

**Fig 2:**
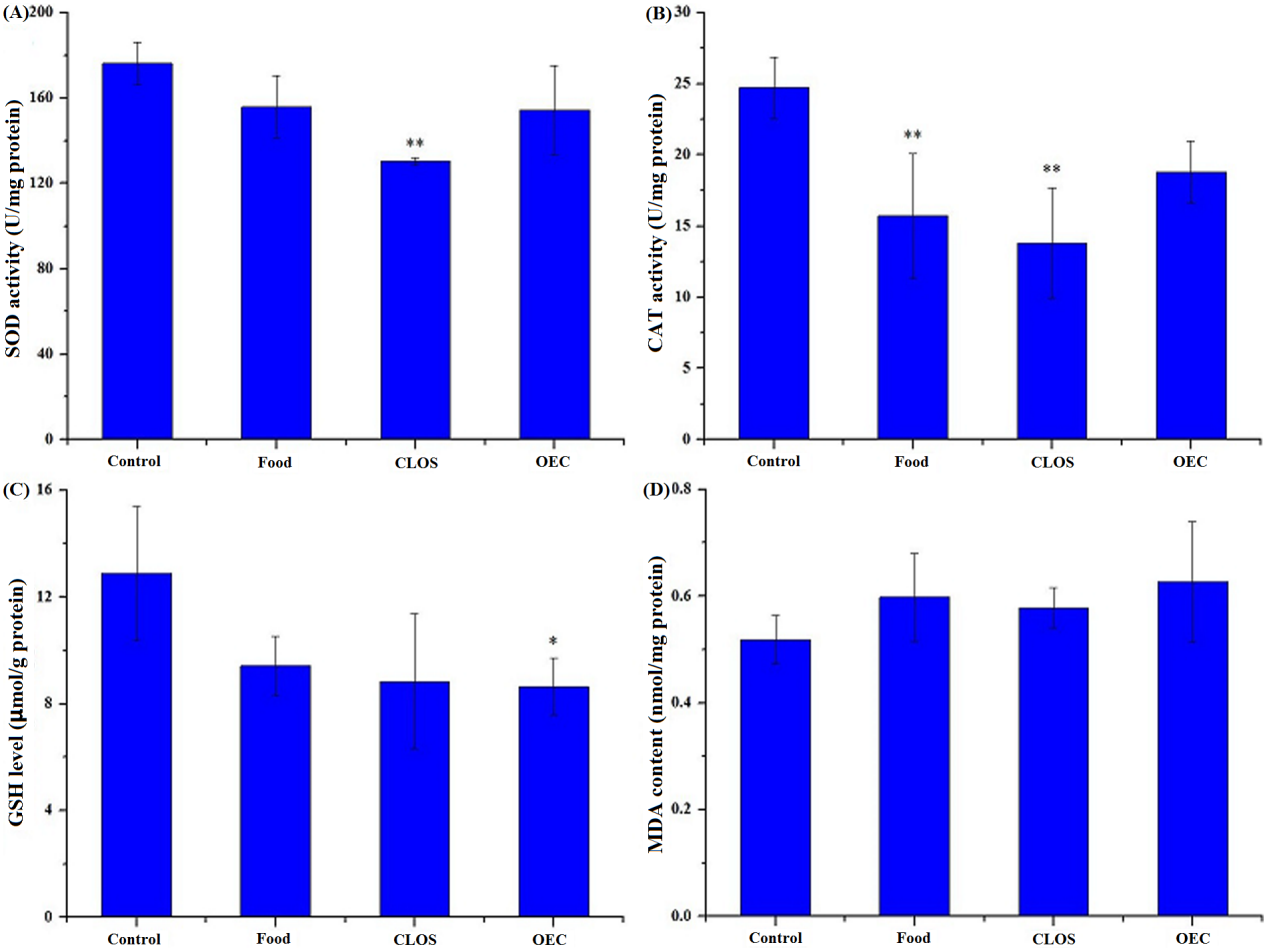
Oxidative stress biomarkers in mice liver after a 24-day exposure to N117 and its combustion products. Antioxidant status was evaluated in mice liver by determining some oxidative stress biomarkers, and these assays were conducted using commercial kits (Nanjing Jiancheng Bioengineering Institute, Nanjing, China). Twenty-four male mice (five-weeks of age) were individually exposed to normal diet (Control), 1/100 wt% N117-treated food (Food), 100 mg N117/L treated by combustion lacking oxygen supplementation (CLOS), and 100 mg N117/L treated by oxygen-enriched combustion (OEC) for 24 days, with six mice in each group. The liver samples were dissected from the mice, homogenized and centrifuged, and the supernatants were collected for biomarker determination. The biochemical parameters for oxidative stress included superoxide dismutase (SOD; A), catalase (CAT; B), reduced glutathione (GSH; C), and the lipid peroxidation product malondialdehyde (MDA; D). Each experiment was performed in triplicate. Data are the mean ± standard deviation (SD), *n* = 6 for each data point. The asterisks indicate significance of differences (*-*p* < 0.05; **-*p* < 0.01) compared with the control using one-way ANOVA with Dunnett’s *t* test.

**Fig 3:**
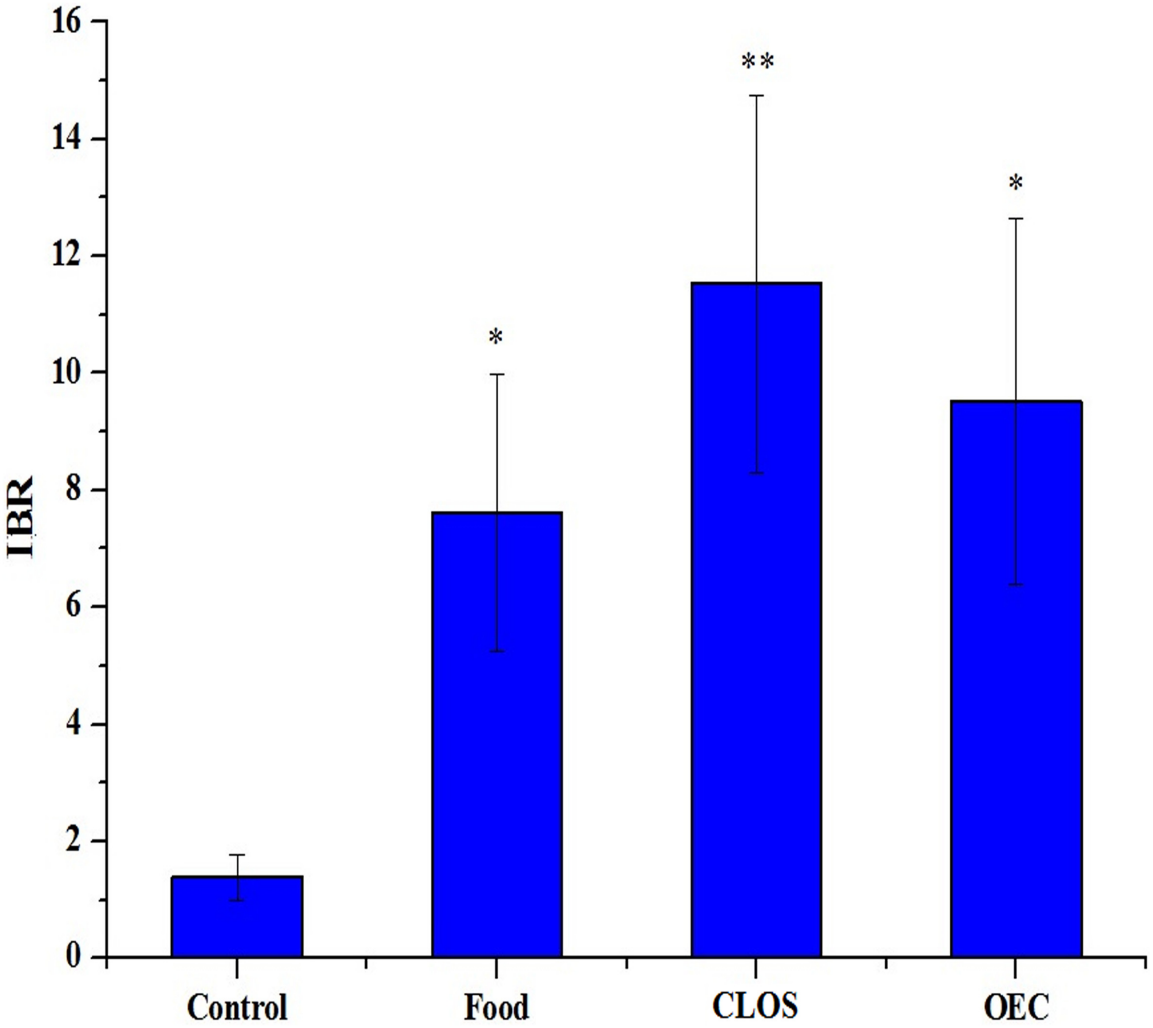
Integrated biomarker response of biochemical parameters in mice liver after a 24-day exposure. Integrated biomarker response (IBR) index was calculated by combining different biomarker signals to provide an overall description of hepatic antioxidant status of male mice (five-weeks of age) individually exposed to normal diet (Control), 1/100 wt% N117-treated food (Food), 100 mg N117/L treated by combustion lacking oxygen supplementation (CLOS), and 100 mg N117/L treated by oxygen-enriched combustion (OEC) for 24 days. The measured oxidative stress biomarkers included superoxide dismutase (SOD), catalase (CAT), reduced glutathione (GSH), and malondialdehyde (MDA). Data are the mean ± standard deviation (SD), *n* = 6 for each data point. The asterisks indicate significance of differences (*-*p* < 0.05; **-*p* < 0.01) compared with the control using one-way ANOVA with Dunnett’s *t* test.

The SOD and CAT activities, GSH content and MDA level in the control and N117-treated groups are shown in Fig 2. Compared to the control, the SOD activity was significantly inhibited (*p* < 0.01) in the CLOS group. Significantly reduced CAT activity (*p* < 0.01) was observed in the Food and CLOS groups, whereas a significant decrease (*p* < 0.05) in the GSH content was detected in the OEC group. No significant increases (*p* > 0.05) were detected for the MDA level in all of the treatment groups. For an overall evaluation of the antioxidant status, these signals were combined into one integral index, termed “IBR”. After these treatments, the IBR values ranged from 0.00 in the control to 5.94 in the CLOS group (Fig 3). The IBR of the treated groups was ranked CLOS > OEC > Food > Control.

### Transcriptome profiles induced by N117 and its combustion products

**Fig 4:**
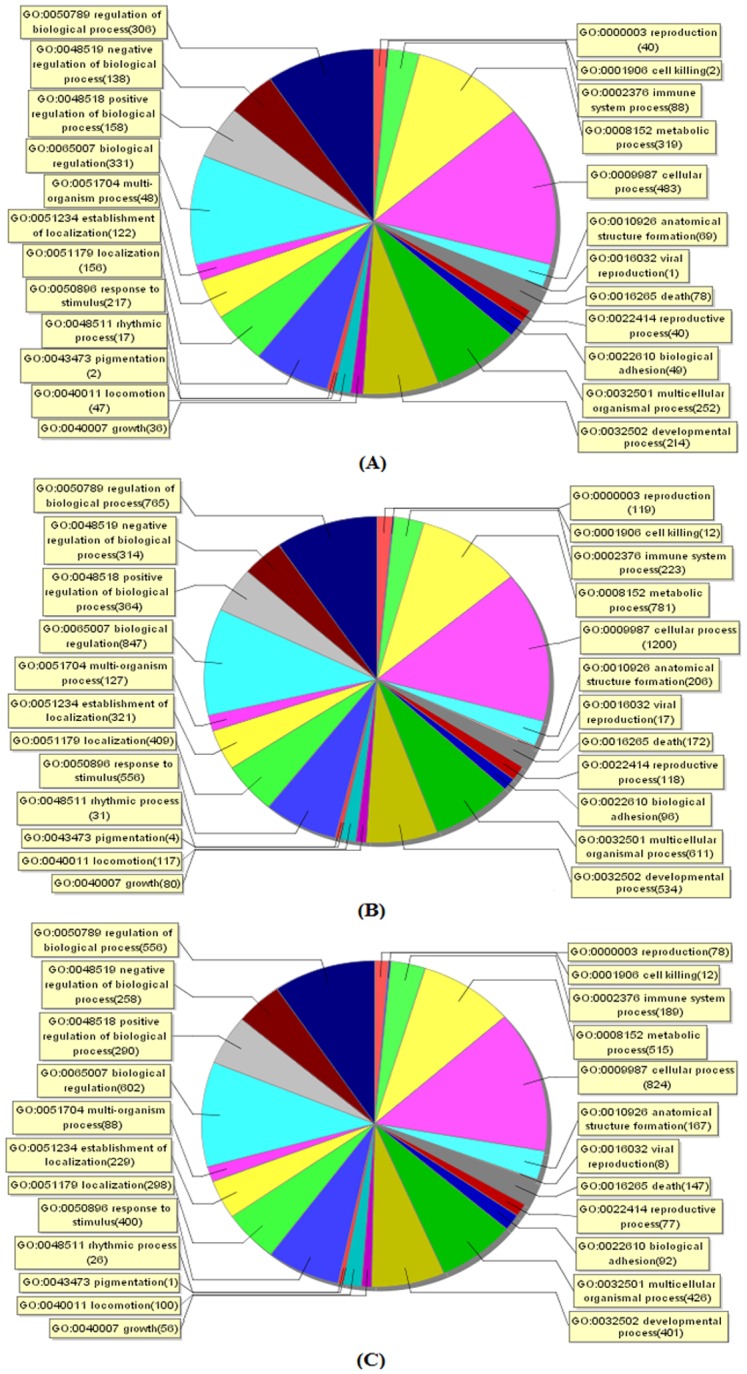
Functional grouping of differentially expressed genes involved in biological processes. Gene expression profiling using Agilent Whole Mouse Genome Oligo Microarray platform containing 41,174 unique probes was performed in male mice (five-weeks of age) individually exposed to normal diet (Control), 1/100 wt% N117-treated food (Food), 100 mg N117/L treated by combustion lacking oxygen supplementation (CLOS), and 100 mg N117/L treated by oxygen-enriched combustion (OEC) for 24 days. RNA extracted from one mouse liver, which was randomly selected in each group, was one sample, and the obtained raw data were normalized with the Quantile algorithm, Gene Spring Software 11.0. Differentially expressed genes (DEGs) involved in biological processes between the treated groups (A, Food; B, CLOS; C, OEC) and control were identified as the genes with a greater than ± 2.0-fold-change and *p*-value < 0.05 (*t*-test). Gene ontology (GO) analysis and Kyoto encyclopedia of genes and genomes (KEGG) pathway analysis were performed using the SBC Analysis System of the Shanghai Biotech Corp.

The transcriptome responses of the mouse livers in the control and three N117-treated groups were analyzed using the Agilent Whole Mouse Genome Oligo Microarray. A total of 1370, 3013, and 2080 genes were identified as DEGs for the Food, CLOS, and OEC groups, respectively. The gene ontology analysis showed that the majority of these DEGs were associated with multiple biological processes, such as cellular process (GO: 0009987), biological regulation (GO: 0065007), and metabolic process (GO: 0008152) (Fig 4).

The biological significance of the DEGs for the different treatments was analyzed using the KEGG pathway database. A KEGG pathway was identified as significantly changed if there were more than three DEGs and the hypergeometric test yielded *p* < 0.05. The significantly altered KEGG pathways are listed in S4 Table and were used for further analysis. These differential pathways were divided into two primary categories, the metabolic pathway and the biological process pathway. Different responses were observed after the different treatments (S4 Table), and the affected pathways involved 6 metabolic pathways and 6 biological processes, including carbohydrate metabolism, lipid metabolism, amino acid metabolism, xenobiotic biodegradation and metabolism, signal transduction, immune system and cellular process. For the Food group, 17 DEGs were involved in the metabolic pathway, including drug metabolism and retinol metabolism (Table 2). The CLOS group had 149 DEGs associated with the metabolic pathway, including drug metabolism, metabolism of xenobiotics by cytochrome P450, glutathione metabolism and retinol metabolism. For the OEC group, drug metabolism and glutathione and arachidonic acid metabolism were the significantly altered metabolic pathways, whereas the related cellular processes included focal adhesion, endocytosis, cell cycle and the p53 signaling pathway (Table 3). Several signal transduction pathways, such as the MARK and TGF-beta signaling pathways, were also significantly affected in the Food and OEC groups (Table 3). In addition, compared with the control, these N117 treatments significantly changed the pathways of immune system and signaling molecules and interaction in mice liver after a 24-day exposure, which mainly included cell adhesion molecules (CAMs), cytokine-cytokine receptor interaction, antigen processing and presentation, and chemokine signaling pathway (Table 4).

**Table 2:** Differentially expressed genes (DEGs) involved in metabolism pathways.

Treatment	Pathway	Count	*p*-Value	Genes
Food	Retinol metabolism	8	0.020	*Cyp26b1; Retsat; Cyp2a2; Cyp26a1; Cyp4a1; Ugt1a2; Cyp3a9; Cyp4a3*
	Drug metabolism	9	0.019	*Cyp2a2; Gstm1; Gsta2; Fmo1; Ugt1a2; Maoa; Gstm3; Cyp3a9; Aox1*
CLOS	Pentose and glucuronate interconversions	9	1.08×10^-4^	*Ugt2b10; Akr1b1; Ugt2b17; Ugdh; Ugt2a3; Ugt2a1; Ugt1a1; Ugt1a2; Ugt2b37; Ugt2b15; Ugt2b*
	Ascorbate and aldarate metabolism	10	4.22×10^-5^	*Ugt2b10; Ugt2b17; Ugdh; Miox; Ugt2a3; Ugt2a1; Gulo; Ugt1a1; Ugt1a2; Ugt2b37; Ugt2b15; Ugt2b*
	Starch and sucrose metabolism	13	7.37×10^-4^	*Ugt2b10; Hk1; Ugt2a3; Agl; Ugt2b15; G6pc; Ugt2b17; Gbe1; Ugdh; Ugt2a1; Gck; Ugt1a2; Ugt1a1; Ugt2b37; Ugt2b*
	Steroid hormone biosynthesis	16	6.38×10^-5^	*Ugt2b10; Hsd3b6; Akr1d1; Hsd3b1; Ugt2a3; Cyp3a9; Ugt2b15; Cyp3a18; Srd5a1; Ugt2b17; Cyp11b3; Ugt2a1; Hsd11b2; Ugt1a2; Ugt1a1; Ugt2b37; Hsd11b1; Ugt2b*
	Glycine, serine and threonine metabolism	13	1.57×10^-4^	*Shmt2; Gatm; Gcat; Tdh; Pipox; Sardh; Phgdh; Alas2; Chdh; Gamt; Gnmt; Sds; Gldc*
	Glutathione metabolism	18	3.06×10^-5^	*Rrm2; Gstt1; Rrm1; RGD1564906; Gpx2; Gsta5; Gsta4; Gclc; RGD1562107; Gstm2; Gpx3; Gstp1; Mgst3; Gstm1; Rrm2b; RGD1559938; Gsta2; Gsto1; Sms; Gstm3*
	Retinol metabolism	17	9.63×10^-4^	*Ugt2b10; Cyp1a2; Cyp2c7; Ugt2a3; Cyp4a1; Cyp3a9; Ugt2b15; Dhrs4; Cyp3a18; Ugt2b17; Cyp2a2; Ugt2a1; Rdh5; Cyp2a3; Ugt1a2; Ugt1a1; Ugt2b37; Cyp4a3; Ugt2b*
	Drug metabolism	29	2.75×10^-9^	*Ugt2b10; Cyp1a2; Cyp2c7; Gstt1; Gsta5; Gsta4; Fmo3; Cyp3a18; Ugt2b17; Mgst3; Ugt1a2; Ugt1a1; Cyp2a3; Ugt2b; RGD1564906; Ugt2a3; Cyp2d4; Cyp3a9; Ugt2b15; RGD1562107; Gstm2; Gstp1; Cyp2a2; Gstm1; Gsta2; Gsto1; Cyp2d5; Ugt2a1; Fmo1; Gstm3; Ugt2b37; Aox1*
	Metabolism of xenobiotics by cytochrome P450	24	1.01×10^-7^	*Ugt2b10; Cyp1a2; Gstt1; Cyp2c7; Gsta5; Gsta4; Cyp3a18; Ugt2b17; Mgst3; Ugt1a1; Ugt1a2; Ugt2b; Ephx1; Cyp2f4; RGD1564906; Ugt2a3; Ugt2b15; Cyp3a9; RGD1562107; Gstm2; Gstp1; Gstm1; Gsta2; Ugt2a1; Gsto1; Ugt2b37; Gstm3*
OEC	Arachidonic acid metabolism	12	0.030	*Cyp2b21; Gpx3; Tbxas1; Ggt6; Gpx2; Cyp2b3; Ggt5; Cyp4f5; Gpx6; Cyp2b2; Cbr3; Ptgs1*
	Glutathione metabolism	13	6.55×10^-4^	*G6pd; Rrm2; Gpx2; Ggt5; Mgst2; RGD1562107; Gstm2; Gpx3; Ggt6; Mgst3; Gsta2; Gpx6; Gstm3*
	Drug metabolism	16	7.35×10^-4^	*Aox2p; Cyp2b3; Fmo3; Fmo5; Mgst2; RGD1562107; Cyp2b21; Gstm2; Cyp2a2; Mgst3; Cyp3a2; Gsta2; Cyp2b2; Fmo1; Cyp2a3; Gstm3*

Values were obtained using Agilent Whole Mouse Genome Oligo Microarray platform on mice liver. Twenty-four male mice (five-weeks of age) were individually exposed to normal diet (Control), 1/100 wt% N117-treated food (Food), 100 mg N117/L treated by combustion lacking oxygen supplementation (CLOS), and 100 mg N117/L treated by oxygen-enriched combustion (OEC) for 24 days, with six mice in each group. Differentially expressed genes (DEGs) involved in metabolism pathways between the treated groups and control were identified as the genes with a greater than ± 2.0-fold-change and *p*-value < 0.05 (*t*-test). Gene ontology (GO) analysis and Kyoto encyclopedia of genes and genomes (KEGG) pathway analysis were performed using the SBC Analysis System of the Shanghai Biotech Corp.

**Table 3:** Differentially expressed genes (DEGs) involved in biological processes (signal transduction and cellular processes).

Treatment	Pathway	Count	*p*-Value	Genes
Food	MARK signaling pathway	20	0.044	*Il1r1; Flnb; Fgf21; Gadd45a; Ddit3; Chp2; Nf1; Dusp2; Nfkb2; Map3k14; Map3k6; Mapk8; Jun; Ntf3; Rela; Il1a; Map3k13; Cd14; Dusp7; Ntrk2*
OEC	TGF-beta signaling pathway	15	0.011	*Tgfb1; Id3; Comp; Zfyve9; Id1; Acvrl1; Lefty2; MGC112830; Amhr2; Amh; Tnf; Inhba; LOC681309; Fst; Tgfb3*
	Endocytosis	25	0.047	*Ret; Adrb2; RT1-EC2; RT1-CE10; RT1-CE16; Arrb2; RT1-CE7; Cxcr4; RT1-CE5; Pdgfra; Psd4; Col20a1; RT1-A3; RT1-CE2; RT1-M3-1; Rab31; RT1-CE4; RT1-Cl; Hspa1a; Ldlr; RT1-S3; RT1-A2; Psd2; Grk5; Mdm2; Sh3kbp1; Met; Csf1r*
	Cell cycle	18	0.032	*Ccnb1; Tgfb1; Gadd45g; Pkmyt1; LOC298795; Cdk1; MGC112830; Mcm5; Espl1; Cdc25c; Ccnb2; Bub1b; Cdkn1a; Mdm2; Mcm2; Wee1; Tgfb3; Cdc20*
	p53 signaling pathway	11	0.046	*Ccnb1; Igfbp3; Gadd45g; LOC298795; Rrm2; Cdk1; Serpine1; Ccnb2; Shisa5; Cdkn1a; Mdm2*
	Focal adhesion	30	0.002	*Comp; Col5a2; Lama1; Mapk8; Actn3; Jun; Col3a1; Vav1; Pdgfra; Col1a1; Fyn; Ppp1cb; Pik3cd; Itga7; Lama2; Pdgfrb; Lamb2; Rac2; Birc3; Vegfa; Itgb7; Prkcb; Vwf; Itga9; Mylk3; Col1a2; Flna; Met; Pak1; LOC681309*

Values were obtained using Agilent Whole Mouse Genome Oligo Microarray platform on mice liver. Twenty-four male mice (five-weeks of age) were individually exposed to normal diet (Control), 1/100 wt% N117-treated food (Food), 100 mg N117/L treated by combustion lacking oxygen supplementation (CLOS), and 100 mg N117/L treated by oxygen-enriched combustion (OEC) for 24 days, with six mice in each group. Differentially expressed genes (DEGs) involved in signal transduction and cellular processes between the treated groups and control were identified as the genes with a greater than ± 2.0-fold-change and *p*-value < 0.05 (*t*-test). Gene ontology (GO) analysis and Kyoto encyclopedia of genes and genomes (KEGG) pathway analysis were performed using the SBC Analysis System of the Shanghai Biotech Corp.

**Table 4:** Differentially expressed genes (DEGs) involved in biological processes (immune system and signaling molecules and interaction).

Treatment	Pathway	Count	*p*-Value	Genes
Food	Cell adhesion molecules (CAMs)	15	0.009	*Jam2; RT1-T24-1; RT1-A3; Cd8a; Cd8b; RT1-M3-1; RT1-EC2; Cldn4; RT1-Cl; RT1-M2; RT1-A2; RT1-CE10; RT1-CE16; RT1-DOa; RT1-Da*
	Antigen processing and presentation	14	2.08×10^-4^	*Nfya; Hspa5; RT1-T24-1; RT1-A3; Cd8a; Cd8b; RT1-M3-1; RT1-EC2; RT1-Cl; RT1-M2; RT1-A2; RT1-CE10; RT1-CE16; RT1-DOa*
CLOS	Cell adhesion molecules (CAMs)	30	0.004	*Cdh4l; Jam2; Cldn14; Mag; Itgal; RT1-EC2; Cd40; Spn; Cd22; RT1-DMa; RT1-CE10; RT1-CE7; RT1-CE16; RT1-DOa; RT1-CE5; Cd226; Cadm3; RT1-DOb; RT1-Bb; RT1-A3; Cd8a; RT1-CE2; Cd8b Sdc3; RT1-Db1; RT1-CE4; RT1-Cl; Itgb7; RT1-S3; RT1-A2*
	Cytokine-cytokine receptor interaction	37	0.004	*Bmpr1a; Cxcr1; Tnfrsf18; Ltb; Csf1; Il10; Il13ra1; Cxcr3; Cd40; Ccl2; Acvr2b; Il1r2; Ccl3; Tnfrsf12a; Lepr; Tnfsf12; Il2rb; Ccl4; Il2ra; Il15; Cxcl2; Il1r1; Relt; Ghr; Acvrl1; Il17ra; Ccl5; Cxcl14; Amh; Tnf; Il1a; Flt3; Tnfrsf14; Il18; Kitlg; Ccr2; Csf1r*
	Antigen processing and presentation	24	1.49×10^-4^	*Psme1; Hspa4; Klrc3; Ifi30; RT1-EC2; RT1-DMa; RT1-CE10; RT1-CE16; RT1-CE7; RT1-DOa; RT1-CE5; Ctss; RT1-DOb; RT1-Bb; Tap1; RT1-A3; Cd8a; RT1-CE2; Cd8b; B2m; RT1-Db1; RT1-CE4; RT1-Cl; RT1-S3*
	Chemokine signaling pathway	33	0.005	*Ccl24; Cxcr1; Gng13; Prkacb; Gng5; Cxcr3; Stat4; Ccl2; Arrb2; Ccl7; Adcy5; Gng12; Ccl3; Rhoc; Vav1; Tiam1; Ccl4; Stat1; Cxcl2; Pik3cd; Xcl1; Rac2; Ccl5; Cxcl14; Prkcb; Ccl9; Rock2; Adcy4; Ccl6; Hck; Pak1; Ccr2; Cxcl1*
OEC	Cell adhesion molecules (CAMs)	29	2.86×10^-5^	*Mag; Itgal; RT1-EC2; Cd40; Spn; Cd22; RT1-DMa; RT1-CE10; RT1-CE7; RT1-CE16; RT1-DOa; RT1-CE5; Cd226; RT1-DOb; RT1-Bb; Cldn11; RT1-A3; Cd8a; RT1-CE2; Cd8b; Sdc3; Cd34; RT1-M3-1; RT1-Db1; RT1-CE4; RT1-Cl; Itgb7; RT1-S3; RT1-A2*
	Cytokine-cytokine receptor interaction	29	0.004	*Tnfrsf25; Tnfrsf21; Tnfrsf18; Il6st; Ltb; Csf1; Il10; Cxcr3; Cd40; Ccl2; Cxcr4; Prlr; Ccl3; Pdgfra; Cxcl2; Relt; Tnfrsf4; Acvrl1; Pdgfrb; Ccl5; Ccr1; Amhr2; Amh; Tnf; Flt3; Tnfrsf14; Met; Ccr2; Csf1r*
	Antigen processing and presentation	25	1.16×10^-7^	*Klrc3; Ifi30; RT1-EC2; RT1-DMa; RT1-CE10; RT1-CE7; RT1-CE16; RT1-DOa; RT1-CE5; Ctss; RT1-DOb; RT1-Bb; Tap1; Tap2; RT1-A3; Cd8a; RT1-CE2; Cd8b; RT1-M3-1; RT1-Db1; RT1-CE4; RT1-Cl; Hspa1a; RT1-S3; RT1-A2*
	Chemokine signaling pathway	26	0.004	*Ccl24; Fgr; Cxcr3; Stat4; Ccl2; Arrb2; Gnb4; Cxcr4; Ccl3; Vav1; Stat1; Cxcl2; Pik3cd; Xcl1; Rac2; Rasgrp2; Ccl5; Ccr1; Prkcb; Ccl9; Grk5; Prex1; Adcy4; Hck; Pak1; Ccr2*

Values were obtained using Agilent Whole Mouse Genome Oligo Microarray platform on mice liver. Twenty-four male mice (five-weeks of age) were individually exposed to normal diet (Control), 1/100 wt% N117-treated food (Food), 100 mg N117/L treated by combustion lacking oxygen supplementation (CLOS), and 100 mg N117/L treated by oxygen-enriched combustion (OEC) for 24 days, with six mice in each group. Differentially expressed genes (DEGs) involved in immune system and signaling molecules and interaction between the treated groups and control were identified as the genes with a greater than ± 2.0-fold-change and *p*-value < 0.05 (*t*-test). Gene ontology (GO) analysis and Kyoto encyclopedia of genes and genomes (KEGG) pathway analysis were performed using the SBC Analysis System of the Shanghai Biotech C

To validate the results of microarray analysis, four DEGs (*Ugt1a2*, *Map3k6*, *Ccnb1* and *Ccl5*) were selected and verified by QRT-PCR. Taken together, the QRT-PCR data closely paralleled the gene expression pattern presented in the microarray data (S5 Table).

## Discussion

The potential toxicity of N117 and its combustion products on mice following a 24-day exposure was assessed in this study using histopathological analysis, biomarker and microarray-based transcriptomics profile analyses. Notable, several significantly altered molecular pathways were identified, suggesting multiple toxicity mechanisms for N117 and its combustion products in mice.

### Histopathological analysis

Histopathological changes indicate cellular damage when organisms are exposed to toxicants. The hepatocellular necrosis and inflammatory infiltration observed in the mouse liver in this study after exposure to the combustion products of N117 indicated their potentials to induce liver injury. As shown in S1 Table and S1 Fig, the possible byproducts under oxygen-lacking conditions included F^-^ and some low-molecular weight organofluorine chemicals, whereas F^-^ was the primary product under the oxygen-enriched conditions. Previous studies reported that F^-^ triggered histopathological alterations in the livers of mice [27] and rats [28] after *in vivo* exposure to different concentrations of sodium fluoride (NaF) in drinking water, which is consistent with the current study of the combustion products of N117.

### Biomarker responses

Under normal conditions, reactive oxygen species (ROS) triggered by xenobiotic exposure are continuously decomposed in cells by antioxidant enzyme defenses, such as SOD and CAT, and low-molecular-mass scavengers, such as GSH [29,30]. Oxidative stress occurs in organisms when the steady-state ROS concentration is transiently or chronically enhanced during toxicant metabolism, leading to the increased damage to different cellular components [31]. In this study, cellular antioxidant defenses were significantly inhibited after the different treatments (SOD for CLOS group, CAT for CLOS and Food groups, and GSH for OEC group; Fig 2). These results suggested that N117 and its combustion products potentially induce oxidative stress in mouse livers. Although the toxicity data for the low-molecular-weight organofluorine compounds in the CLOS group are currently lacking, F^-^-induced oxidative stress in rats or mice has been previously reported [32–34]. However, our data did not show any significant alteration in the MDA content after these various treatments, indicating the absence of lipid peroxidation during the 24-day exposure.

The IBR index was used to reflect the overall stress of the different treatments. This approach provides a simple but powerful tool for comparing the biological effects by combining different biomarker signals [25] and has been commonly used in recent environmental risk assessment studies [26,35]. The IBR index showed that the CLOS group exhibited a higher stress than the OEC group. This is most likely because the CLOS products contained both F^-^ and low-molecular-weight organofluorine compounds, whereas the OEC products were primarily composed of F^-^. In addition, the IBR value for the Food group was higher than the control, suggesting the possible toxic effects of N117 via direct ingestion.

### Transcriptomics analysis

After the 24-day exposure to three different treatments of N117, the altered KEGG pathways in the mouse liver were divided into two main categories, the metabolic pathway and biological process pathway. In this study, three major metabolic pathways, xenobiotic biodegradation and metabolism, carbohydrate metabolism, and lipid metabolism, and four biological processes, signal transduction, cellular processes, immune system, and signaling molecules and interaction, were selected for further analysis.

Xenobiotic biodegradation and metabolism was the most affected metabolic pathway for all three treatments, with 9, 53, and 16 DEGs in the Food, CLOS, and OEC groups, respectively. The majority of the DEGs involved in the detoxification of xenobiotics were associated with the aberrant regulation of cytochrome P450 enzymes (CYPs) and glutathione S-transferases (GSTs) [36], which is consistent with earlier studies of exposure of other toxicants [37,38]. As biomarkers for the toxicity of drugs and xenobiotics, CYPs and GSTs have been widely used to assess the potential toxicity of environmental pollutants [39,40]. Carbohydrate metabolism related pathways were also altered in the livers of the CLOS group, including pentose and glucuronate interconversion (9 DEGs), ascorbate and aldarate metabolism (10 DEGs), and starch and sucrose metabolism (13 DEGs). Of these DEGs, the down-regulated genes, including *ugt1a1*, *ugt1a2*, *ugt2a1*, *ugt2a3*, *ugt2b*, *ugt2b10*, *ugt2b15*, *ugt2b17*, and *ugt2b37*, were related to the regulation of UDP-glucuronosyltransferases (UGTs). Microsomal UGTs, primarily expressed in mammalian liver, are a family of isoenzymes that catalyze the transfer of UDP-glucuronic acid to endogenous and exogenous chemicals and/or their metabolites, rendering these substances more polar and facilitating their excretion into the bile and urine [41–43]. The alterations of the expression of these genes suggested that the combustion products of N117 under oxygen-lacking conditions might change the carbohydrate metabolism in the mouse liver at transcriptome level. In addition, for lipid metabolism, variations in the steroid hormone biosynthesis (16 DEGs) and arachidonic acid metabolism (12 DEGs) were identified in the CLOS and OEC groups. These observations indicate that the N117 combustion products can disrupt lipid metabolism in mouse livers.

The microarray analysis also showed some significant alterations in signal transduction pathways, including the MARK signaling pathway (20 DEGs) in the Food group and the TGF-beta signaling pathway (15 DEGs) in the OEC group. The MARK signaling pathway is involved in various cellular functions, including cell proliferation, differentiation and migration, and couples intracellular responses to growth factor binding to cell surface receptors [44,45]. Aberrance of this pathway has been implicated in several human diseases, such as inflammation and cancers [36]. By contrast, the TGF-beta system functions via protein kinase receptors and Smad mediators to regulate many biological processes, including morphogenesis, embryonic development, immune regulation, wound healing and inflammation [46,47]. Alterations of the TGF-beta signaling pathway are involved in a broad range of pathologies, such as cancer, cardiovascular pathology, fibrosis and congenital diseases [46,48]. The DEGs in these two pathways were primarily down-regulated in this study, and the disruption of these DEGs demonstrates that N117 and its combustion products potentially affect the signal transduction pathways in mouse livers during a 24-day exposure.

For the cellular processes, several altered pathways were identified in the OEC group, including focal adhesion (30 DEGs), endocytosis (25 DEGs), cell cycle (18 DEGs), and the p53 signaling pathway (11 DEGs). These results indicate that the combustion products of N117 under oxygen-enriched conditions induce cytotoxicity in mouse livers, which may be caused by F^-^ toxicity because F^-^ is the primary component in the OEC products. Previous studies have also suggested the F^-^-induced cytotoxicity occurs in mouse embryonic stem cells [49] and cultured mouse osteoblasts [50]. Furthermore, the hepatocellular necrosis and inflammatory infiltration detected in our histological examination also reflected the toxic effects on cellular processes.

Moreover, these N117 treatments induced several significantly changed pathways of immune system and signaling molecules and interaction, such as CAMs, cytokine-cytokine receptor interaction, antigen processing and presentation, and chemokine signaling pathway. Interestingly, in this work, a subset of the DEGs identified in GO analysis (*Ccl2*, *Ccl3*, *Ccl5*, *Ccr2* and *Cxcl2*) comprises part of the cytokine-cytokine receptor interaction and chemokine signaling pathway, which play a role in health and are essential in the communication between cells in the immune system [51]. These genes have been previously indicated to modulate the immunological and inflammatory responses in disease [52,53]. Besides, CAMs, which are important in a variety of physiological and pathological conditions related to interactions between cells and in measuring the specificity of cell-cell binding [54], were also significantly altered in mice liver after N117 treatments, indicating their possible toxic effects on this pathway.

## Conclusions

Our data indicated that N117 and its combustion products induce histopathological damage and oxidative stress in mouse livers during the experimental exposure. Microarray-based transcriptomics profiling showed that these N117 treatments mainly disrupted the metabolism of xenobiotics, carbohydrates and lipids as well as some biological processes, including signal transduction, cellular processes, immune system, and signaling molecules and interaction. This is the first toxicity study to simulate mouse exposure to N117 and its combustion products via waste disposal processes, and these alarming results necessitate additional studies to evaluate the long-term toxic effects and elucidate the underlying cellular and molecular mechanisms of N117 toxicity on living organisms.

## Supporting Information

S1 ARRIVE Guidelines Checklist (DOC)

S1 Fig The total ion current (TIC) chromatograph (A1-D1) obtained by liquid chromatography coupled with a high-resolution hybrid quadrupole time-of-flight mass spectrometry (LCMS-Q-TOF) and the corresponding mass spectrum (retention time: 0.714–11.302 min) (A2-D2) subtracted by the mass spectrum (retention time: 0.323–0.610 min). (A) the pure water, (B) the absorption solution of N117 treated by combustion lacking oxygen supplementation (CLOS), (C) the absorption solution of filter paper using the oxygen flask combustion (OFC) method, (D) the absorption solution of N117 treated by oxygen-enriched combustion (OEC). Chromatographic separation was performed at a flow rate of 250 μL/min using a Thermo BDS Hypersil C_18_ column (2.1 mm × 100 mm, particle size 2.4 μm) maintained at 40°C. The mobile phase was 0.3% formic acid in water and acetonitrile with an isocratic elution of 40:60 (v/v). Injection volume was 10 μL and elution time was 20 min for all samples. Mass spectrometric analysis was carried out with a Q-TOF MS operating in a negative ion mode using an electrospray ion source.
(DOC)

S1 File The experimental procedures of the oxygen flask combustion (OFC) method. (DOC)

S2 File Ion chromatography analysis for drinking water samples. (DOC)

S3 File LCMS-Q-TOF analysis for different combustion products of N117. (DOC)

S4 File The calculation procedures of the integrated biomarker response (IBR) index. (DOC)

S5 File The experimental procedures of quantitative real-time polymerase chain reaction (QRT-PCR) analysis. (DOC)

S1 Table The thermal degradation products of perfluorosulfonic acid copolymer. (DOC)

S2 Table Gene-specific primer sequences used for QRT-PCR. (DOC)

S3 Table The body weight (BW) and relative organ weight of mice in control and N117-treated groups after 24 days. (DOC)

S4 Table Categories of significantly altered KEGG pathways affected by N117 and its combustion products. (DOC)

S5 Table QRT-PCR validation of changes in selected genes identified by microarrays. (DOC)

## References

[1] Devanathan R (2008) Recent developments in proton exchange membranes for fuel cells. Energy & Environmental Science 1: 101–119.

[2] Iwai Y, Hiroki A, Tamada M, Yamanishi T (2008) Radiation deterioration in mechanical properties and ion exchange capacity of Nafion N117 swelling in water. Journal of Membrane Science 322: 249–255.

[3] Cele N, Ray SS (2009) Recent progress on Nafion-based nanocomposite membranes for fuel cell applications. Macromolecular Materials and Engineering 294: 719–738.

[4] Lee HJ, Cho MK, Jo YY, Lee KS, Kim HJ, Cho E, et al (2012) Application of TGA techniques to analyze the compositional and structural degradation of PEMFC MEAs. Polymer Degradation and Stability 97: 1010–1016.

[5] Mauritz KA, Moore RB (2004) State of understanding of Nafion. Chemical Reviews 104: 4535–4585. 15669162 10.1021/cr0207123

[6] Chen C, Levitin G, Hess DW, Fuller TF (2007) XPS investigation of Nafion membrane degradation. Journal of Power Sources 169: 288–295.

[7] Kurniawan D, Arai H, Morita S, Kitagawa K (2013) Chemical degradation of Nafion ionomer at a catalyst interface of polymer electrolyte fuel cell by hydrogen and oxygen feeding in the anode. Microchemical Journal 106: 384–388.

[8] DuPont Fuel Cells (2009) Safe Handling and Use of Perfluorosulfonic Acid Products (Technical Information).

[9] Sugawara T, Kawashima N, Murakami TN (2011) Kinetic study of Nafion degradation by Fenton reaction. Journal of Power Sources 196: 2615–2620.

[10] Yu TH, Sha Y, Liu WG, Merinov BV, Shirvanian P, Goddard WA (2011) Mechanism for degradation of Nafion in PEM fuel cells from quantum mechanics calculations. Journal of the American Chemical Society 133: 19857–19863. 10.1021/ja2074642 22017316

[11] Qiu YY, Qu XJ, Dong J, Ai SY, Han RX (2011) Electrochemical detection of DNA damage induced by acrylamide and its metabolite at the graphene-ionic liquid-Nafion modified pyrolytic graphite electrode. Journal of Hazardous Materials 190: 480–485. 10.1016/j.jhazmat.2011.03.071 21497017

[12] Yeh MH, Sun CL, Su JS, Lin LY, Lee CP, Chen CY, et al (2012) A low-cost counter electrode of ITO glass coated with a graphene/Nafion composite film for use in dye-sensitized solar cells. Carbon 50: 4192–4202.

[13] Lien HL, Zhang WX (2007) Removal of methyl *tert*-butyl ether (MTBE) with Nafion. Journal of Hazardous Materials 144: 194–199. 17110027 10.1016/j.jhazmat.2006.10.004

[14] Nasef MM, Yahaya AH (2009) Adsorption of some heavy metal ions from aqueous solutions on Nafion 117 membrane. Desalination 249: 677–681.

[15] Lettieri T (2006) Recent applications of DNA microarray technology to toxicology and ecotoxicology. Environmental Health Perspectives 114: 4–9. 16393650 10.1289/ehp.8194 PMC1332648

[16] Wei YH, Liu Y, Wang JS, Tao Y, Dai JY (2008) Toxicogenomic analysis of the hepatic effects of perfluorooctanoic acid on rare minnows (*Gobiocypris rarus*). Toxicology and Applied Pharmacology 226: 285–297. 17976672 10.1016/j.taap.2007.09.023

[17] Wang FQ, Liu W, Jin YH, Dai JY, Yu WG, Liu XH, et al (2010) Transcriptional effects of prenatal and neonatal exposure to PFOS in developing rat brain. Environmental Science & Technology 44: 1847–1853. 20136073 10.1021/es902799f

[18] Geng WH, Nakajima T, Takanashi H, Ohki A (2007) Determination of total fluoride in coal by use of oxygen flask combustion method with catalyst. Fuel 86: 715–721.

[19] Kobayashi Y, Tian ML, Eguchi M, Mallouk TE (2009) Ion-exchangeable, electronically conducting layered perovskite oxyfluorides. Journal of the American Chemical Society 131: 9849–9855. 10.1021/ja9040829 19548670

[20] Oberley LW, Spitz DR (1984) Assay of superoxide dismutase activity in tumor tissue. Methods in Enzymology 105: 457–464. 6547201 10.1016/s0076-6879(84)05064-3

[21] Goth L (1991) A simple method for determination of serum catalase activity and revision of reference range. Clinica Chimica Acta 196: 143–151. 2029780 10.1016/0009-8981(91)90067-m

[22] Redegeld FAM, van Opstal MAJ, Houdkamp E, van Bennekom WP (1988) Determination of glutathione in biological materials by flow-injection analysis using an enzymatic recycling reaction. Analytical Biochemistry 174: 489–495. 2977066 10.1016/0003-2697(88)90048-6

[23] Yagi K (1998) Simple assay for the level of total lipid peroxides in serum or plasma. Methods in Molecular Biology 108: 101–106. 9921519 10.1385/0-89603-472-0:101

[24] Bradford MM (1976) A rapid and sensitive method for the quantitation of microgram quantities of protein utilizing the principle of protein-dye binding. Analytical Biochemistry 72: 248–254. 942051 10.1016/0003-2697(76)90527-3

[25] Beliaeff B, Burgeot T (2002) Integrated biomarker response: A useful tool for ecological risk assessment. Environmental Toxicology and Chemistry 21: 1316–1322. 12069320

[26] Li ZH, Velisek J, Zlabek V, Grabic R, Machova J, Kolarova J, et al (2011) Chronic toxicity of verapamil on juvenile rainbow trout (*Oncorhynchus mykiss*): Effects on morphological indices, hematological parameters and antioxidant responses. Journal of Hazardous Materials 185: 870–880. 10.1016/j.jhazmat.2010.09.102 20970250

[27] Chattopadhyay A, Podder S, Agarwal S, Bhattacharya S (2011) Fluoride-induced histopathology and synthesis of stress protein in liver and kidney of mice. Archives of Toxicology 85: 327–335. 10.1007/s00204-010-0588-7 20859737

[28] Chouhan S, Lomash V, Flora SJS (2010) Fluoride-induced changes in haem biosynthesis pathway, neurological variables and tissue histopathology of rats. Journal of Applied Toxicology 30: 63–73. 10.1002/jat.1474 19743388

[29] Cederbaum AI, Lu YK, Wu DF (2009) Role of oxidative stress in alcohol-induced liver injury. Archives of Toxicology 83: 519–548. 10.1007/s00204-009-0432-0 19448996

[30] Li ZH, Li P, Rodina M, Randak T (2010) Effect of human pharmaceutical Carbamazepine on the quality parameters and oxidative stress in common carp (*Cyprinus carpio* L.) spermatozoa. Chemosphere 80: 530–534. 10.1016/j.chemosphere.2010.04.046 20466407

[31] Lushchak VI (2011) Environmentally induced oxidative stress in aquatic animals. Aquatic Toxicology 101: 13–30. 10.1016/j.aquatox.2010.10.006 21074869

[32] Shanthakumari D, Srinivasalu S, Subramanian S (2004) Effect of fluoride intoxication on lipidperoxidation and antioxidant status in experimental rats. Toxicology 204: 219–228. 15388248 10.1016/j.tox.2004.06.058

[33] Chouhan S, Flora SJS (2008) Effects of fluoride on the tissue oxidative stress and apoptosis in rats: Biochemical assays supported by IR spectroscopy data. Toxicology 254: 61–67. 10.1016/j.tox.2008.09.008 18845224

[34] Kanbur M, Eraslan G, Silici S, Karabacak M (2009) Effects of sodium fluoride exposure on some biochemical parameters in mice: Evaluation of the ameliorative effect of royal jelly applications on these parameters. Food and Chemical Toxicology 47: 1184–1189. 10.1016/j.fct.2009.02.008 19425189

[35] Wang C, Lu GH, Wang PF, Wu H, Qi PD, Liang Y (2011) Assessment of environmental pollution of Taihu Lake by combining active biomonitoring and integrated biomarker response. Environmental Science & Technology 45: 3746–3752. 21413737 10.1021/es1037047

[36] Ahlborn GJ, Nelson GM, Ward WO, Knapp G, Allen JW, Ouyang M, et al (2008) Dose response evaluation of gene expression profiles in the skin of K6/ODC mice exposed to sodium arsenite. Toxicology and Applied Pharmacology 227: 400–416. 10.1016/j.taap.2007.10.029 18191166

[37] Keshava C, Divi RL, Einem TL, Richardson DL, Leonard SL, Keshava N, et al (2009) Chlorophyllin significantly reduces benzo[a]pyrene-DNA adduct formation and alters cytochrome P450 1A1 and 1B1 expression and EROD activity in normal human mammary epithelial cells. Environmental and Molecular Mutagenesis 50: 134–144. 10.1002/em.20449 19152381 PMC2637934

[38] Zhang Y, Zhang ZY, Zhao YP, Cheng SP, Ren HQ (2013) Identifying health effects of exposure to trichloroacetamide using transcriptomics and metabonomics in mice (*Mus musculus*). Environmental Science & Technology 47: 2918–2924. 23406383 10.1021/es3048976

[39] Li XC, Schuler MA, Berenbaum MR (2007) Molecular mechanisms of metabolic resistance to synthetic and natural xenobiotics. Annual Review of Entomology 52: 231–253. 16925478 10.1146/annurev.ento.51.110104.151104

[40] Xie YX, Liu J, Benbrahim-Tallaa L, Ward JM, Logsdon D, Diwan BA, et al (2007) Aberrant DNA methylation and gene expression in livers of newborn mice transplacentally exposed to a hepatocarcinogenic dose of inorganic arsenic. Toxicology 236: 7–15. 17451858 10.1016/j.tox.2007.03.021 PMC2465467

[41] Tephly TR, Burchell B (1990) UDP-glucuronosyltransferases: a family of detoxifying enzymes. Trends in Pharmacological Sciences 11: 276–279. 2117826 10.1016/0165-6147(90)90008-v

[42] Matkovic LB, D’Andrea F, Fornes D, San Martín de Viale LC, Mazzetti MB (2011) How porphyrinogenic drugs modeling acute porphyria impair the hormonal status that regulates glucose metabolism. Their relevance in the onset of this disease. Toxicology 290: 22–30. 10.1016/j.tox.2011.08.014 21889565

[43] Rowland A, Miners JO, Mackenzie PI (2013) The UDP-glucuronosyltransferases: Their role in drug metabolism and detoxification. International Journal of Biochemistry & Cell Biology 45: 1121–1132. 23500526 10.1016/j.biocel.2013.02.019

[44] Iida C, Fujii K, Kishioka T, Nagae R, Onishi Y, Ichi I, et al (2007) Activation of mitogen activated protein kinase (MARK) during carbon tetrachloride intoxication in the rat liver. Archives of Toxicology 81: 489–493. 17285312 10.1007/s00204-007-0181-x

[45] Wu B, Liu S, Guo XC, Zhang Y, Zhang XX, Li M, et al (2012) Responses of mouse liver to dechlorane plus exposure by integrative transcriptomic and metabonomic studies. Environmental Science & Technology 46: 10758–10764. 22913625 10.1021/es301804t

[46] Santibañez JF, Quintanilla M, Bernabeu C (2011) TGF-*β*/TGF-β receptor system and its role in physiological and pathological conditions. Clinical Science 121: 233–251. 10.1042/CS20110086 21615335

[47] Song YJ, Li J, Xie XF, Wang H, Li QX (2011) Effects of amlodipine on TGF-β-induced Smad2, 4 expressions in adriamycin toxicity of rat mesangial cells. Archives of Toxicology 85: 663–668. 10.1007/s00204-011-0667-4 21337027

[48] Moustakas A, Heldin CH (2009) The regulation of TGFβ signal transduction. Development 136: 3699–3714. 10.1242/dev.030338 19855013

[49] Ngoc TDN, Son YO, Lim SS, Shi XL, Kim JG, Heo JS, et al (2012) Sodium fluoride induces apoptosis in mouse embryonic stem cells through ROS-dependent and caspase- and JNK-mediated pathways. Toxicology and Applied Pharmacology 259: 329–337. 10.1016/j.taap.2012.01.010 22285274 PMC3299821

[50] Wang Z, Yang XY, Yang SY, Ren GX, Ferreri M, Su Y, et al (2011) Sodium fluoride suppress proliferation and induce apoptosis through decreased insulin-like growth factor-I expression and oxidative stress in primary cultured mouse osteoblasts. Archives of Toxicology 85: 1407–1417. 10.1007/s00204-011-0697-y 21461751

[51] Gervin K, Vigeland MD, Mattingsdal M, Hammerø M, Nygård H, Olsen AO, et al (2012) DNA methylation and gene expression changes in monozygotic twins discordant for psoriasis: identification of epigenetically dysregulated genes. PLoS Genetics 8: e1002454 10.1371/journal.pgen.1002454 22291603 PMC3262011

[52] Turrin NP, Plata-Salamán CR (2000) Cytokine-cytokine interactions and the brain. Brain Research Bulletin 51: 3–9. 10654575 10.1016/s0361-9230(99)00203-8

[53] Scott K, Manunta M, Germain C, Smith P, Jones M, Mitchell P, et al (2005) Qualitatively distinct patterns of cytokines are released by human dendritic cells in response to different pathogens. Immunology 116: 245–254. 16162273 10.1111/j.1365-2567.2005.02218.x PMC1817823

[54] Krieglstein CF, Granger DN (2001) Adhesion molecules and their role in vascular disease. American Journal of Hypertension 14: 44S–54S. 11411765 10.1016/s0895-7061(01)02069-6

