# Supplementary material for: Hepatic Transcriptome Responses in Mice (Mus musculus) Exposed to the Nafion Membrane and Its Combustion Products
Source: PLoS One. 2015 Jun 9;10(6):e0128591. doi: 10.1371/journal.pone.0128591 (PMC4461320; doi:10.1371/journal.pone.0128591)
Supplement: S1 Fig — (A) the pure water, (B) the absorption solution of N117 treated by combustion lacking oxygen supplementation (CLOS), (C) the absorption solution of filter paper using the oxygen flask combustion (OFC) method, (D) the absorption solution of N117 treated by oxygen-enriched combustion (OEC). Chromatographic separation was performed at a flow rate of 250 μL/min using a Thermo BDS Hypersil C18 column (2.1 mm × 100 mm, particle size 2.4 μm) maintained at 40°C. The mobile phase was 0.3% formic acid in water and acetonitrile with an isocratic elution of 40:60 (v/v). Injection volume was 10 μL and elution time was 20 min for all samples. Mass spectrometric analysis was carried out with a Q-TOF MS operating in a negative ion mode using an electrospray ion source. (DOC) [file pone.0128591.s002.doc]

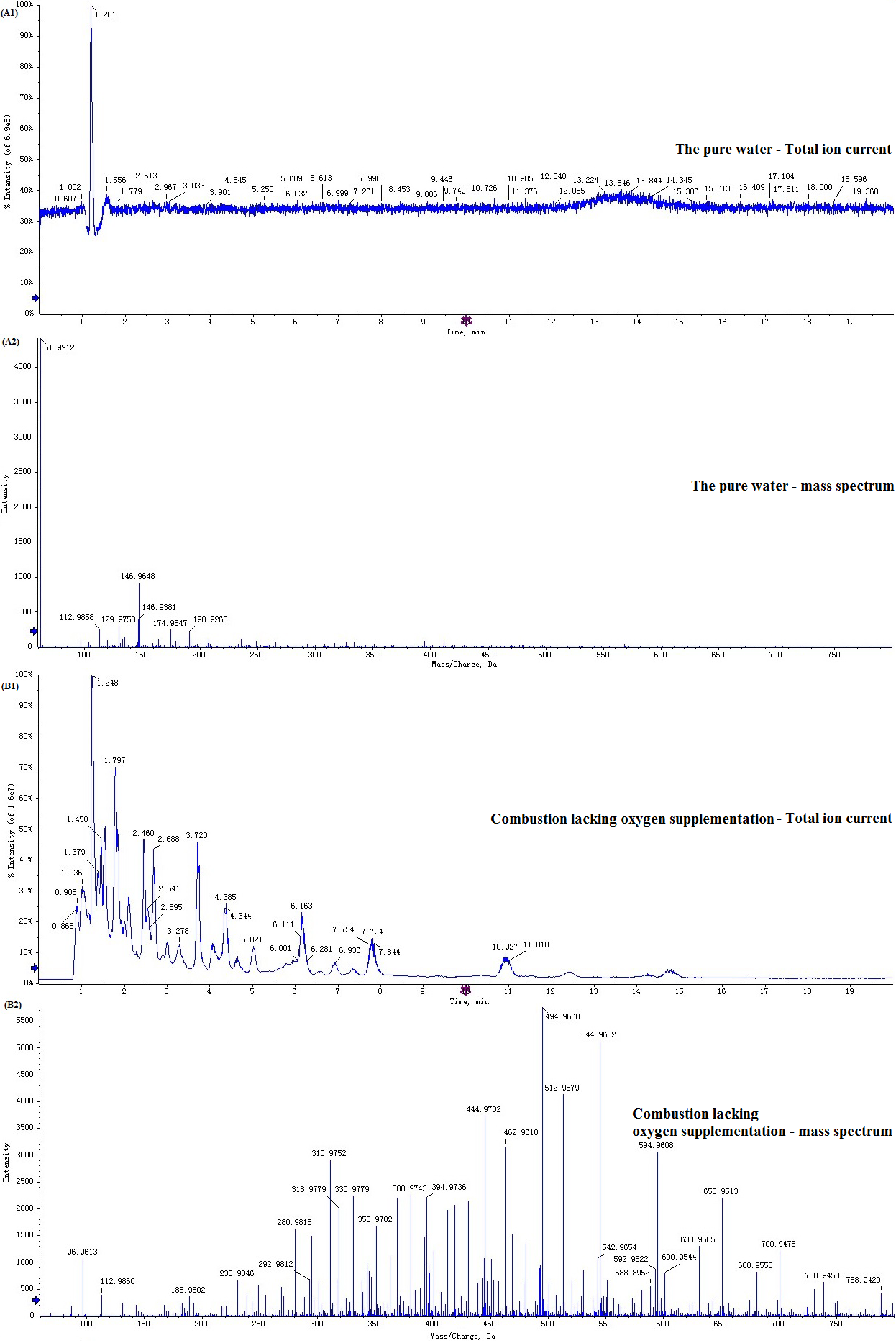


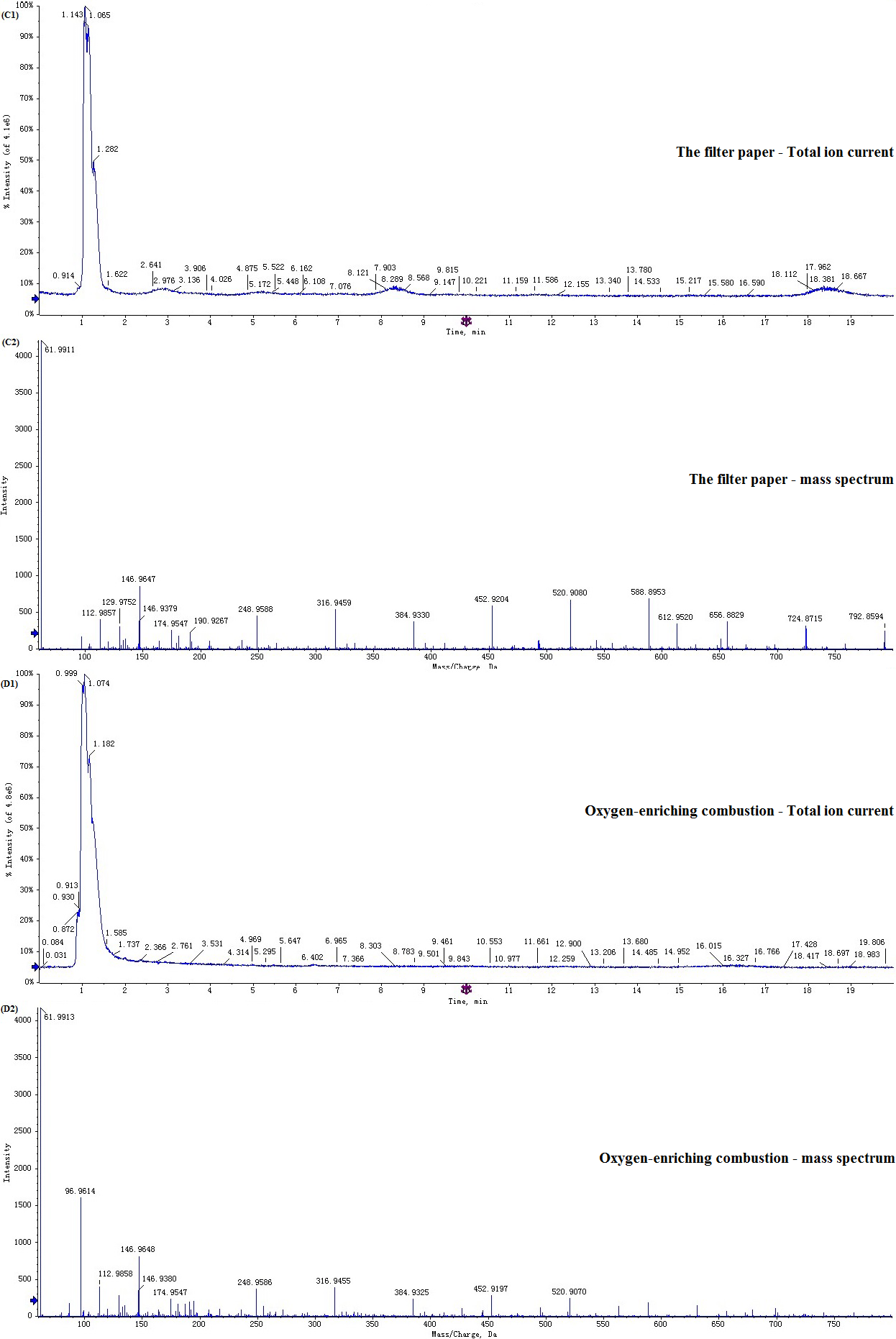


**S1 Fig.** **The total ion current (TIC) chromatograph (A1-D1) obtained by liquid chromatography coupled with a high-resolution hybrid quadrupole time-of-flight mass spectrometry (LCMS-Q-TOF) and the corresponding mass spectrum (retention time: 0.714-11.302 min) (A2-D2) subtracted by the mass spectrum (retention time: 0.323-0.610 min).** (A) the pure water, (B) the absorption solution of N117 treated by combustion lacking oxygen supplementation (CLOS), (C) the absorption solution of filter paper using the oxygen flask combustion (OFC) method, (D) the absorption solution of N117 treated by oxygen-enriched combustion (OEC). Chromatographic separation was performed at a flow rate of 250 μL/min using a Thermo BDS Hypersil C_18_ column (2.1 mm × 100 mm, particle size 2.4 μm) maintained at 40 ℃. The mobile phase was 0.3% formic acid in water and acetonitrile with an isocratic elution of 40:60 (v/v). Injection volume was 10 μL and elution time was 20 min for all samples. Mass spectrometric analysis was carried out with a Q-TOF MS operating in a negative ion mode using an electrospray ion source.
