# Supplementary material for: Hepatic Transcriptome Responses in Mice (Mus musculus) Exposed to the Nafion Membrane and Its Combustion Products
Source: PLoS One. 2015 Jun 9;10(6):e0128591. doi: 10.1371/journal.pone.0128591 (PMC4461320; doi:10.1371/journal.pone.0128591)
Supplement: S1 File — (DOC) [file pone.0128591.s003.doc]

**S1 File.** **The experimental procedures of the oxygen flask combustion (OFC) method.**

0.05 g small-sized N117 were precisely weighed, and transferred on a 40 × 40 (mm) ash-free filter paper. After folded into a cylindrical shape, the paper with N117 samples was inserted into the platinum cage attached to the flask plug. The fuse was ignited, and the samples were combusted in the 250 mL oxygen-filled flask containing 50 mL NaOH solution (0.05 M). After the combustion, the flask was shaken for 2 min, and allowed to stand for 1 h. The procedure of this method can also be clearly illustrated in Fig. 1 of the literature [1] and cited as below.


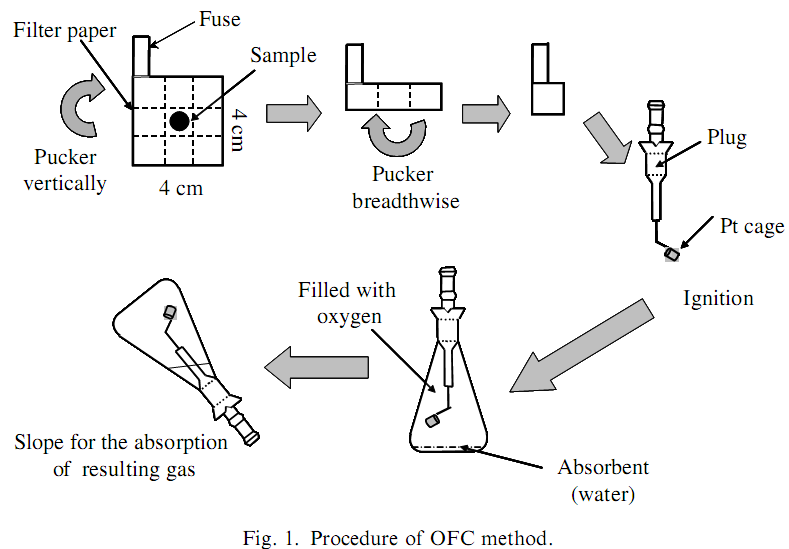


Supplementary References

Geng WH, Nakajima T, Takanashi H, Ohki A (2007) Determination of total fluoride in coal by use of oxygen flask combustion method with catalyst. Fuel 86: 715-721.
