# Supplementary material for: Hepatic Transcriptome Responses in Mice (Mus musculus) Exposed to the Nafion Membrane and Its Combustion Products
Source: PLoS One. 2015 Jun 9;10(6):e0128591. doi: 10.1371/journal.pone.0128591 (PMC4461320; doi:10.1371/journal.pone.0128591)
Supplement: S2 File — (DOC) [file pone.0128591.s004.doc]

**S2 File. Ion chromatography analysis for drinking water samples.**

F^-^ concentrations in the exposure solutions were quantified using an ion-chromatograph system (ICS-1000, Dionex, USA). The system was equipped with an autosampler (sample injection volume: 10 μL), a pump, a degasser, a guard column, and a separation column (Dionex IonPac AS 12A, 4 mm i.d × 200 mm, USA) operating at 30 ℃. The mobile phase was 20 mM KOH and the flow rate was set at 1.0 mL/min. F^-^ was quantified by external calibration. A range of calibration curve was 1-20 mg/L (r^2^ = 0.9995).
