# Supplementary material for: Hepatic Transcriptome Responses in Mice (Mus musculus) Exposed to the Nafion Membrane and Its Combustion Products
Source: PLoS One. 2015 Jun 9;10(6):e0128591. doi: 10.1371/journal.pone.0128591 (PMC4461320; doi:10.1371/journal.pone.0128591)
Supplement: S3 File — (DOC) [file pone.0128591.s005.doc]

**S3 File. LCMS-Q-TOF analysis for different combustion products of N117.**

The initial absorbed solutions samples of different combustion methods (CLOS and OEC) were filtered through a 0.22 μm membrane filter and subjected to the LCMS-Q-TOF analysis. A pure water sample and an absorbed solution of only filter paper using the OFC method were individually treated and analyzed to deduct the background of the former two N117 samples. Chromatographic separation was performed at a flow rate of 250 μL/min using a Thermo BDS Hypersil C_18_ column (2.1 mm × 100 mm, particle size 2.4 μm) (Thermo Fisher Scientific, Waltham, MA) maintained at 40 ℃. The mobile phase was 0.3% formic acid in water (A) and acetonitrile (B) with an isocratic elution of 40:60 (v/v). Injection volume was 10 μL and elution time was 20 min for all samples. Mass spectrometric analysis was carried out with a Q-TOF MS (Triple TOF 5600, AB SCIEX) operating in a negative ion mode using an electrospray ion source. Mass range of TOF MS was m/z 60-800. The other experiment parameters were set as follows: curtain gas, 35 (arbitrary units); ion source gas 1, 55 (arbitrary units); ion source gas 2, 55 (arbitrary units); temperature, 550 ℃; ionspray voltage floating, -4500 kV; declustering potential, -80 V; collision energy, -10 eV. The high-resolution LC-MS data was acquired using Analyst TF 1.6 (AB SCIEX) and processed using PeakView 1.2 (AB SCIEX).
