# Supplementary material for: Hepatic Transcriptome Responses in Mice (Mus musculus) Exposed to the Nafion Membrane and Its Combustion Products
Source: PLoS One. 2015 Jun 9;10(6):e0128591. doi: 10.1371/journal.pone.0128591 (PMC4461320; doi:10.1371/journal.pone.0128591)
Supplement: S4 File — (DOC) [file pone.0128591.s006.doc]

**S4 File. The calculation procedures of the integrated biomarker response (IBR) index.**

For each biomarker: (1) Calculation of mean and SD for each treatment. (2) Standardization of data for each treatment: *F_i_’* = (*F_i_* – mean *F*)/*S*, where *F_i_’* is the standardized value of the biomarker, *F_i_* is the specific value of a biomarker from each treatment, mean *F* is the mean of the biomarker calculated for all treatments, and *S* is the standard deviation calculated for the treatment-specific values of each biomarker. (3) Using standardized data, *Z* was computed as +*F_i_’* in the case of activation and –*F_i_’* in the case of an inhibition, and then the minimum value for all treatments for each biomarker was obtained and added to *Z*. Finally, the score *B* was computed as *B* = |min *F_i_’*| + *Z*, where *B* ≥ 0 and |min *F_i_’*| is the absolute value of minimum *F_i_’*. For all the biomarkers treated this way: calculation of star plot areas by multiplication of the obtained value of each biomarker (*B_i_*) with the value of the next biomarker, arranged as a set, dividing each calculation by 2 and summing-up of all values. The corresponding IBR value is {[(*B_1_*×*B_2_*)/2] + [(*B_2_*×*B_3_*)/2] + … + [(*B_n-1_*×*B_n_*)/2] + [(*B_n_*×*B_1_*)/2]}.
