# Supplementary material for: Hepatic Transcriptome Responses in Mice (Mus musculus) Exposed to the Nafion Membrane and Its Combustion Products
Source: PLoS One. 2015 Jun 9;10(6):e0128591. doi: 10.1371/journal.pone.0128591 (PMC4461320; doi:10.1371/journal.pone.0128591)
Supplement: S5 File — (DOC) [file pone.0128591.s007.doc]

**S5 File. The experimental procedures of quantitative real-time polymerase chain reaction (QRT-PCR) analysis.**

Four genes (*Ugt1a2*, *Map3k6*, *Ccnb1* and *Ccl5*) were selected from DEGs in the microarray analysis for QRT-PCR verification. Briefly, cDNA was synthesized from the total RNA samples in each treatment, using the First Strand cDNA Synthesis Kit (TaKaRa, Japan). The forward and reverse primer sequences for these selected genes were presented in Table S2. QRT-PCR analysis was performed using the Power SYBR Green PCR Master Mix (Applied Biosystems, Foster City, CA, USA), while PCR amplification and cDNA quantification were conducted using an ABI PRISM 7000 Sequence Detection System (Applied Biosystems, Foster City, CA, USA). The relative differences in expression between groups were expressed using cycle time (C_t_) values: the C_t_ values of the interested genes were firstly normalised with *β*-actin of the same sample, and then the relative differences between control and treated groups were calculated and expressed as relative increases, setting control as 100%.
