# Supplementary material for: Hepatic Transcriptome Responses in Mice (Mus musculus) Exposed to the Nafion Membrane and Its Combustion Products
Source: PLoS One. 2015 Jun 9;10(6):e0128591. doi: 10.1371/journal.pone.0128591 (PMC4461320; doi:10.1371/journal.pone.0128591)
Supplement: S1 Table — (DOC) [file pone.0128591.s008.doc]

**S1 Table. The thermal degradation products of perfluorosulfonic acid copolymer [1].**

| Compound | Evolution Temp., °C | mg/g Sample |
| --- | --- | --- |
| SO_2_ | 280 | 15 |
| CO_2_ | 300 | 30 |
| HF | 400 | -^*^ |
| CO | 400 | 3 |
| R_f_COF | 400 | 10^**^ |
| COF_2_ | 400 | 3 |
| COS | 400 | Trace |
| R_f_OH | 400 | Trace |

Values were obtained by DuPont using the Standard Infrared Analysis of Thermal Effluents (IRATE) technique. The possible chemical compositions of the effluent from perfluorinated sulfonic acid copolymer were measured at the following conditions: the atmosphere was air, and the flow rate was 13 mL/min. The sample was heated in a stainless steel tube at 10 °C/min to 200 °C and then for 5 °C/min to 400 °C; the temperature was then held for an additional 20 minutes for a total run time of approximately 75 minutes. ^*^ Significant level but concentration could not be determined because HF reacts with and absorbs on cell walls. ^**^ Mixture of products.

Supplementary References

1. DuPont Fuel Cells (2009) Safe Handling and Use of Perfluorosulfonic Acid Products (Technical Information).
