# Supplementary material for: Hepatic Transcriptome Responses in Mice (Mus musculus) Exposed to the Nafion Membrane and Its Combustion Products
Source: PLoS One. 2015 Jun 9;10(6):e0128591. doi: 10.1371/journal.pone.0128591 (PMC4461320; doi:10.1371/journal.pone.0128591)
Supplement: S2 Table — (DOC) [file pone.0128591.s009.doc]

**S2 Table. Gene-specific primer sequences used for QRT-PCR.**

| Gene symbol | Forward sequence (5’ to 3’) | Reverse sequence (5’ to 3’) | Product size (bp) |
| --- | --- | --- | --- |
| *Ugt1a2* | ACTCGGGCATTCATCAC | TCACCATCGGAACTCCA | 73 |
| *Map3k6* | AGGGCGGCTACCTCAAGAT | TGTGAACGATACGGTTCTCATG | 168 |
| *Ccnb1* | CGTAGACGCAGATGATGG | CACGACCCTGTAGGTATTTT | 122 |
| *Ccl5* | CACCACTCCCTGCTGCTT | ACTTGGCGGTTCCTTCG | 129 |
