# Supplementary material for: Hepatic Transcriptome Responses in Mice (Mus musculus) Exposed to the Nafion Membrane and Its Combustion Products
Source: PLoS One. 2015 Jun 9;10(6):e0128591. doi: 10.1371/journal.pone.0128591 (PMC4461320; doi:10.1371/journal.pone.0128591)
Supplement: S3 Table — (DOC) [file pone.0128591.s010.doc]

**S3 Table. The body weight (BW) and relative organ weight of mice in control and N117-treated groups after 24 days.**

|  | Control | Food | CLOS | OEC |
| --- | --- | --- | --- | --- |
| Body weight (g) | 40.51±2.57 | 40.20±3.81 | 39.21±3.49 | 41.93±2.38 |
| Liver (mg/100 g BW) | 5442±729 | 5179±328 | 6113±452 | 5658±269 |
| Kidney (mg/100 g BW) | 1674±187 | 1569±277 | 1622±158 | 1551±196 |

Values were obtained by determining the body weight and relative organ weight of mice tissues (liver and kidney). Twenty-four male mice (five-weeks of age) were individually exposed to normal diet (Control), 1/100 wt% N117-treated food (Food), 100 mg N117/L treated by combustion lacking oxygen supplementation (CLOS), and 100 mg N117/L treated by oxygen-enriched combustion (OEC) for 24 days, with six mice in each group. After the exposure, all mice after diethyl ether anaesthesia were sacrificed by cervical dislocation for tissue collection. Data are the mean ± standard deviation (SD), *n* = 6 for each data point. The significance of differences between the treated groups and control was analyzed using one-way ANOVA with Dunnett’s *t* test.
