# Supplementary material for: Hepatic Transcriptome Responses in Mice (Mus musculus) Exposed to the Nafion Membrane and Its Combustion Products
Source: PLoS One. 2015 Jun 9;10(6):e0128591. doi: 10.1371/journal.pone.0128591 (PMC4461320; doi:10.1371/journal.pone.0128591)
Supplement: S4 Table — (DOC) [file pone.0128591.s011.doc]

**S4 Table. Categories of significantly altered KEGG pathways affected by N117 and its combustion products.**

| Treatment | Category | Pathway Name | Pathway ID | Count | *p*-Value |
| --- | --- | --- | --- | --- | --- |
| **Food** | **Metabolism** |  |  |  |  |
|  | Metabolism of cofactors and vitamins | Retinol metabolism | rno00830 | 8 | 0.020 |
|  | Xenobiotics biodegradation and metabolism | Drug metabolism | rno00982 | 9 | 0.019 |
|  | **Biological process** |  |  |  |  |
|  | Signal transduction | MARK signaling pathway | rno04010 | 20 | 0.044 |
|  | Signaling molecules and interaction | Cell adhesion molecules (CAMs) | rno04514 | 15 | 0.009 |
|  | Immune system | Hematopoietic cell lineage | rno04640 | 12 | 9.51×10^-4^ |
|  |  | Toll-like receptor signaling pathway | rno04620 | 10 | 0.025 |
|  |  | Antigen processing and presentation | rno04612 | 14 | 2.08×10^-4^ |
|  | | | | | |
| **CLOS** | **Metabolism** |  |  |  |  |
|  | Carbohydrate metabolism | Pentose and glucuronate interconversions | rno00040 | 9 | 1.08×10^-4^ |
|  |  | Ascorbate and aldarate metabolism | rno00053 | 10 | 4.22×10^-5^ |
|  |  | Starch and sucrose metabolism | rno00500 | 13 | 7.37×10^-4^ |
|  | Lipid metabolism | Steroid hormone biosynthesis | rno00140 | 16 | 6.38×10^-5^ |
|  | Amino acid metabolism | Glycine, serine and threonine metabolism | rno00260 | 13 | 1.57×10^-4^ |
|  | Metabolism of other amino acids | Glutathione metabolism | rno00480 | 18 | 3.06×10^-5^ |
|  | Metabolism of cofactors and vitamins | Retinol metabolism | rno00830 | 17 | 9.63×10^-4^ |
|  | Xenobiotics biodegradation and metabolism | Drug metabolism | rno00982 | 29 | 2.75×10^-9^ |
|  |  | Metabolism of xenobiotics by cytochrome P450 | rno00980 | 24 | 1.01×10^-7^ |
|  | **Biological process** |  |  |  |  |
|  | Signaling molecules and interaction | Cell adhesion molecules (CAMs) | rno04514 | 30 | 0.004 |
|  |  | Cytokine-cytokine receptor interaction | rno04060 | 37 | 0.004 |
|  | Immune system | Hematopoietic cell lineage | rno04640 | 24 | 1.86×10^-5^ |
|  |  | Complement and coagulation cascades | rno04610 | 18 | 0.002 |
|  |  | Antigen processing and presentation | rno04612 | 24 | 1.49×10^-4^ |
|  |  | Leukocyte transendothelial migration | rno04670 | 22 | 0.026 |
|  |  | Intestinal immune network for IgA production | rno04672 | 12 | 0.013 |
|  |  | Chemokine signaling pathway | rno04062 | 33 | 0.005 |
|  | | | | | |
| **OEC** | **Metabolism** |  |  |  |  |
|  | Lipid metabolism | Arachidonic acid metabolism | rno00590 | 12 | 0.030 |
|  | Metabolism of other amino acids | Glutathione metabolism | rno00480 | 13 | 6.55×10^-4^ |
|  | Xenobiotics biodegradation and metabolism | Drug metabolism | rno00982 | 16 | 7.35×10^-4^ |
|  | **Biological process** |  |  |  |  |
|  | Signal transduction | TGF-beta signaling pathway | rno04350 | 15 | 0.011 |
|  | Signaling molecules and interaction | Cytokine-cytokine receptor interaction | rno04060 | 29 | 0.004 |
|  |  | ECM-receptor interaction | rno04512 | 17 | 9.14×10^-4^ |
|  |  | Cell adhesion molecules (CAMs) | rno04514 | 29 | 2.86×10^-5^ |
|  | Transport and catabolism | Endocytosis | rno04144 | 25 | 0.047 |
|  | Cell growth and death | Cell cycle | rno04110 | 18 | 0.032 |
|  |  | p53 signaling pathway | rno04115 | 11 | 0.046 |
|  | Cell communication | Focal adhesion | rno04510 | 30 | 0.002 |
|  | Immune system | Hematopoietic cell lineage | rno04640 | 19 | 5.48×10^-5^ |
|  |  | Toll-like receptor signaling pathway | rno04620 | 14 | 0.036 |
|  |  | NOD-like receptor signaling pathway | rno04621 | 11 | 0.032 |
|  |  | Natural killer cell mediated cytotoxicity | rno04650 | 18 | 0.035 |
|  |  | Antigen processing and presentation | rno04612 | 25 | 1.16×10^-7^ |
|  |  | T cell receptor signaling pathway | rno04660 | 18 | 0.008 |
|  |  | B cell receptor signaling pathway | rno04662 | 15 | 0.003 |
|  |  | Leukocyte transendothelial migration | rno04670 | 18 | 0.014 |
|  |  | Intestinal immune network for IgA production | rno04672 | 13 | 2.28×10^-4^ |
|  |  | Chemokine signaling pathway | rno04062 | 26 | 0.004 |

Values were obtained using Agilent Whole Mouse Genome Oligo Microarray platform on mice liver. Twenty-four male mice (five-weeks of age) were individually exposed to normal diet (Control), 1/100 wt% N117-treated food (Food), 100 mg N117/L treated by combustion lacking oxygen supplementation (CLOS), and 100 mg N117/L treated by oxygen-enriched combustion (OEC) for 24 days, with six mice in each group. Differentially expressed genes (DEGs) between the treated groups and control were identified as the genes with a greater than ± 2.0-fold-change and *p*-value < 0.05 (*t*-test). Gene ontology (GO) analysis and Kyoto encyclopedia of genes and genomes (KEGG) pathway analysis were performed using the SBC Analysis System of the Shanghai Biotech Corp.
