# Supplementary material for: Hepatic Transcriptome Responses in Mice (Mus musculus) Exposed to the Nafion Membrane and Its Combustion Products
Source: PLoS One. 2015 Jun 9;10(6):e0128591. doi: 10.1371/journal.pone.0128591 (PMC4461320; doi:10.1371/journal.pone.0128591)
Supplement: S5 Table — (DOC) [file pone.0128591.s012.doc]

**S5 Table. QRT-PCR validation of changes in selected genes identified by microarrays.**

| Symbol | Gene name | **Microarray** | | | **QRT-PCR (*n* = 6)** | | |
| --- | --- | --- | --- | --- | --- | --- | --- |
|  |  | Food | CLOS | OEC | Food | CLOS | OEC |
| *Ugt1a2* | UDP glucuronosyltransferase 1 family, polypeptide A2 | -2.13 | -4.21 | -1.11 | -2.68 ± 0.53 | -5.62 ± 0.34 | -1.51 ± 0.14 |
| *Map3k6* | mitogen-activated protein kinase kinase kinase 6 | -3.42 | 4.29 | -2.44 | -2.93 ± 0.42 | 5.33 ± 0.71 | -3.95 ± 0.27 |
| *Ccnb1* | cyclin B1 | -2.15 | -5.20 | -3.20 | -3.98 ± 0.25 | -6.86 ± 0.45 | -4.58 ± 0.79 |
| *Ccl5* | chemokine (C-C motif) ligand 5 | -1.19 | -8.09 | -6.88 | -2.25 ± 0.24 | -7.78 ± 0.58 | -5.33 ± 0.49 |

Confirmation of four selected genes (*Ugt1a2*, *Map3k6*, *Ccnb1* and *Ccl5*) from the microarrays was performed by using QRT-PCR. Values were obtained by the relative expression compared to the control and expressed as fold change. Twenty-four male mice (five-weeks of age) were individually exposed to normal diet (Control), 1/100 wt% N117-treated food (Food), 100 mg N117/L treated by combustion lacking oxygen supplementation (CLOS), and 100 mg N117/L treated by oxygen-enriched combustion (OEC) for 24 days, with six mice in each group. Differentially expressed genes (DEGs) between the treated groups and control were identified as the genes with a greater than ± 2.0-fold-change and *p*-value < 0.05 (*t*-test).
